# Biophysical Screening Pipeline for Cryo-EM Grid Preparation of Membrane Proteins

**DOI:** 10.3389/fmolb.2022.882288

**Published:** 2022-06-23

**Authors:** Stephan Niebling, Katharina Veith, Benjamin Vollmer, Javier Lizarrondo, Osvaldo Burastero, Janina Schiller, Angelica Struve García, Philipp Lewe, Carolin Seuring, Susanne Witt, María García-Alai

**Affiliations:** ^1^ European Molecular Biology Laboratory Hamburg, Hamburg, Germany; ^2^ Centre for Structural Systems Biology (CSSB), Hamburg, Germany; ^3^ Centre for Structural Systems Biology (CSSB), Leibniz Institute of Virology (LIV), Hamburg, Germany; ^4^ Centre for Structural Systems Biology (CSSB), University Medical Center Hamburg-Eppendorf, Hamburg, Germany; ^5^ Centre for Structural Systems Biology (CSSB), Department of Chemistry, University of Hamburg, Hamburg, Germany

**Keywords:** mass photometry, differential scanning fluorimetry (DSF), membrane proteins, dynamic light scattering, sample preparation, electron cryo-microscopy (cryo-EM), biophysical characterization

## Abstract

Successful sample preparation is the foundation to any structural biology technique. Membrane proteins are of particular interest as these are important targets for drug design, but also notoriously difficult to work with. For electron cryo-microscopy (cryo-EM), the biophysical characterization of sample purity, homogeneity, and integrity as well as biochemical activity is the prerequisite for the preparation of good quality cryo-EM grids as these factors impact the result of the computational reconstruction. Here, we present a quality control pipeline prior to single particle cryo-EM grid preparation using a combination of biophysical techniques to address the integrity, purity, and oligomeric states of membrane proteins and its complexes to enable reproducible conditions for sample vitrification. Differential scanning fluorimetry following the intrinsic protein fluorescence (nDSF) is used for optimizing buffer and detergent conditions, whereas mass photometry and dynamic light scattering are used to assess aggregation behavior, reconstitution efficiency, and oligomerization. The data collected on nDSF and mass photometry instruments can be analyzed with web servers publicly available at spc.embl-hamburg.de. Case studies to optimize conditions prior to cryo-EM sample preparation of membrane proteins present an example quality assessment to corroborate the usefulness of our pipeline.

## Introduction

Since the resolution revolution in cryo-EM, it became evident that high-quality samples are required to arrive at high-resolution structures (Kühlbrandt, 2014; Joppe et al., 2020). Obtaining good quality frozen-hydrated samples suitable for the study by cryo-EM can be challenging, even when starting from highly purified homogeneous protein solutions (Glaeser, 2021). Some common problems encountered when moving from soluble to vitrified samples include unexplained aggregation (D'Imprima et al., 2019), disintegrated particles and loss of subunits from complexes, crowded coated or low number of particles found within holes, and particles adopting a preferential orientation (Tan et al., 2017). These problems are often caused by the tendency of particles to adsorb to the air–water interface, a problem widely discussed in the literature for cryo-EM (Noble et al., 2018; D'Imprima et al., 2019). The effect of the interaction with the air–water interface can be mitigated by the addition of mild detergents like fluorinated octyl maltoside (Efremov et al., 2015), but will not be further addressed in this article.

To identify optimal vitrification parameters, it is often necessary to try a variety of experimental conditions, following an iterative process, before arriving at optimal thin aqueous ice films on grids. Importantly, the use of protein samples that vary in quality from batch-to-batch purification should be prevented during grid optimization. Addressing the sample quality control (QC) of proteins is not trivial; however, guidelines have been established for the improvement of research data reproducibility (Passmore and Russo, 2016; de Marco et al., 2021). Addressing the quality of membrane protein samples is even more challenging, which usually makes their structural determination difficult. Efforts have been made with respect to the expression of membrane proteins in mammalian systems (Goehring et al., 2014) and initial quality control steps focusing on screening strategies prior to protein purification (Nji et al., 2018; Chatzikyriakidou et al., 2021). Structural methods investigating membrane proteins require the extraction and solubilization from the membrane using detergents, and the use of glycerol gradient centrifugation has proven to be efficient for the mild removal of free detergent prior to cryo-EM studies (Hauer et al., 2015).

**FIGURE 1:**
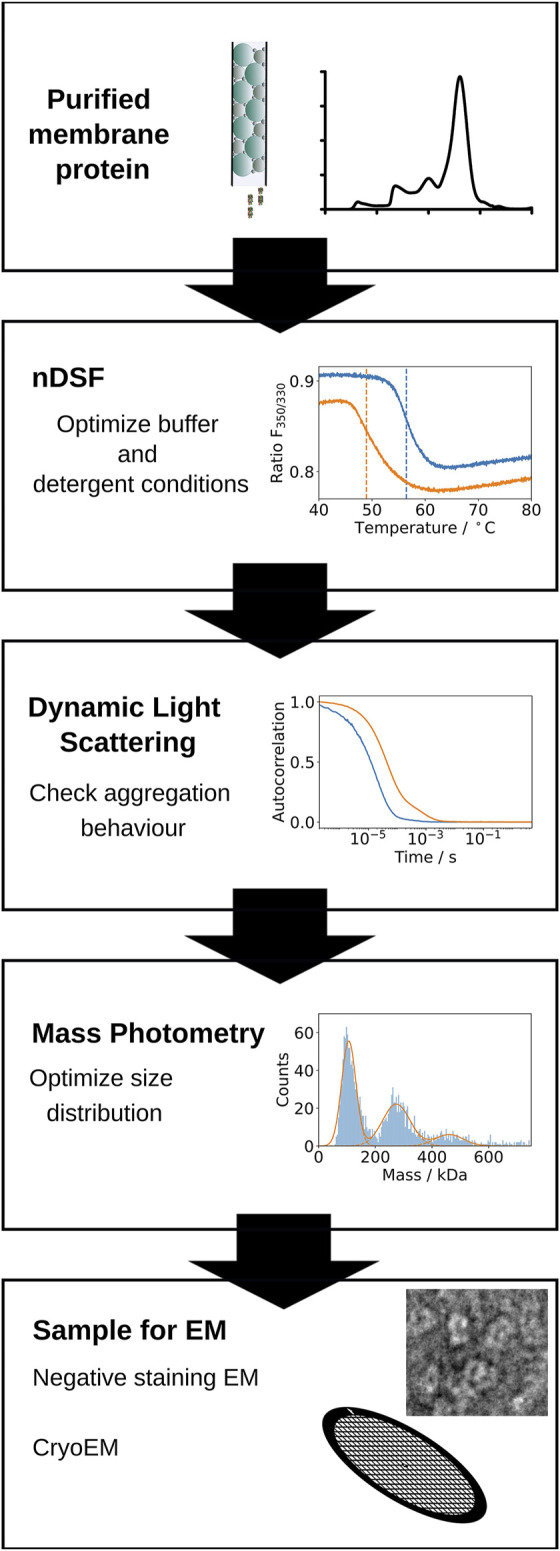
Schematic representation of the screening pipeline proposed. First, optimal buffer and detergent conditions for the stability of a purified membrane protein sample are identified by nDSF. Selected conditions are used for further screening by Dynamic Light Scattering (DLS) to determine the aggregation status of the protein under the specific buffer and detergent conditions. Mass photometry measurements are used to confirm the expected molecular weight of the protein and the distribution of masses present in the sample. Finally, the sample is imaged successively by negative stain and cryo-EM.

Within this protocol, we introduce a simple pipeline for benchmarking the quality of purified membrane protein samples prior to their vitrification. Purified and biophysically characterized proteins would be the starting material to obtain well-dispersed particles on grids for high resolution cryo-EM single particle structural studies (Figure 1). Membrane protein sample optimization has indeed several bottlenecks throughout the sample preparation process. A crucial step would be probing protein stability after detergent solubilization (Kotov et al., 2019). Here, we apply differential scanning fluorimetry (nDSF) in combination with static light scattering upon thermal denaturation and dynamic light scattering (DLS) to optimize buffer/detergent selection and to minimize the aggregation of membrane protein samples. The evaluation of the particle size and distribution in the reconstituted sample is further characterized by mass photometry (MP) and negative-stain transmission electron microscopy (negative-stain EM). nDSF allows the study of the unfolding of membrane proteins, following the intrinsic fluorescence of tryptophan residues during a thermal ramp in different buffers and detergents. In addition to the calculation of the melting temperature, the device follows the onset of aggregation by monitoring static light scattering (Kotov et al., 2019). DLS reports on the polydispersity or aggregational state of a membrane protein in solution (Murphy, 1997; Raynal et al., 2014). Particles of different sizes move, creating flickering, and all the motions and measurements are described by auto-correlation functions. The technique provides a batch average of spherical modelled particles in suspension, and only those populations for which hydrodynamic radii differ by a factor of 3 are efficiently resolved. Finally, MP linearly correlates the interference of the scattered light of single particles landing on the measuring surface with their mass, allowing the characterization of mass distributions of macromolecules in solution (Young et al., 2018; Hundt, 2021). Furthermore, the quantification of complex assembly formation (Häußermann et al., 2019; Soltermann et al., 2020; Wu and Piszczek, 2020) and the characterization of membrane proteins using different solubilization approaches has successfully been applied (Olerinyova et al., 2020; Heermann et al., 2021; Steiert et al., 2022). Advantages of this technique are the low sample-consumption and the simple and fast measurement (Wu and Piszczek, 2021). The working concentration is in the 100 nM range (a few microliters needed), which is close to concentrations used for negative-stain EM (Lai et al., 2021) and measurements are performed typically in 60 s. It is therefore a powerful screening tool to characterize protein samples before electron microscopy (Sonn-Segev et al., 2020). In addition, we developed an online tool for analyzing the results of mass photometry experiments. This new module, called PhotoMol, is freely available in the eSPC data analysis platform (spc.embl-hamburg.de).

After confirming the mass distribution of the sample by mass photometry, our quality control pipeline (Figure 1) foresees a visual inspection of the sample by negative-stain EM, a technique where the sample is embedded in a film of heavy atoms (Hall 1955; Brenner and Horne, 1959). There are a lot of excellent descriptions on how to prepare negatively stained samples for electron microscopy (Brenner and Horne, 1959; Horne et al., 1975) or in the form of cryo-negative staining (Adrian et al., 1998). Amongst the more recent protocols, Scarff et al. outline the potential pitfalls and available workarounds (Scarff et al., 2018). Generally, negative-stained samples are fairly easy to prepare and can give valuable insights on sample morphology, protein homogeneity, and dispersity of particles on EM grids including aggregation and particle concentration early on. In the past, images of well-stained, evenly dispersed particles have been used to create a first, low resolution (∼17 Å) reconstruction (De Rosier and Klug 1968; Ohi et al., 2004; Gallagher et al., 2019), which can be used as initial model in later processing steps of cryo-EM sample images. Also, the conditions (buffer composition, protein concentration) found to be suitable for negative stain sample preparation can inform about the concentrations necessary for cryo-EM samples, which are usually a factor of 10 higher. Our standard protocol for negative stain is described in the “Stepwise Procedures” section and uses 2–20 µM protein concentration and 2% uranyl acetate solution as stain for a first sample quality assessment.

While negative stain is suited to characterize rigid, globular proteins, for membrane proteins, it does not necessarily allow conclusions to be drawn whether the protein is amenable to high-resolution studies by cryo-EM (Hoenger and Aebi, 1996). For the latter, parameters including protein concentration, buffer (Drulyte et al., 2018), and grid type as well as the solubilization method using detergents, amphipols, or nanodiscs have to be optimized and are determined best directly from vitrified cryo-EM specimens. Glow-discharging parameters are also strongly affecting the ice layer and sample dispersion. The preparation of vitrified EM specimens is outlined in the “Stepwise Procedures” section.

Sample preparation of cryo-EM grids for membrane proteins remains, and will remain, mostly an empirical and iterative process for each specific target. However, there is literature available that helps in the trouble-shooting, providing systematic investigations for buffer, blotting, and grid type selection (Kampjut et al., 2021). Here, we are presenting a biophysical pipeline to be used for challenging samples and complexes, prior to cryo-EM experiments, where having a better characterized sample can be advantageous during the grid optimization stage.

## Materials and Equipment

### Protein Production and Purification

**IJ1**: The IJ1 protein was expressed in *E.coli* LEMO21 cells and ZY-autoinduction media with an addition of 0.3 mM rhamnose. Cultures were grown to an OD_600nm_ of 1 and cooled down to 20°C, induced with 0.1 mM isopropyl-β-d-1-thiogalactopyranoside (IPTG) and left for expression overnight. Cells were collected by centrifuging 25 min at 5,000 × g and pellets were stored at −20°C. To obtain the membrane fraction, cells were resuspended in 2 ml/g pellets with buffer [30 mM tris(hydroxymethyl)aminomethane hydrochloride (Tris-HCl) pH 7.5, 250 mM NaCl, 10% glycerol, 200 μg/ml DNaseI (AppliChem), 2 mM MgCl_2_ and 200 μg/ml Lysozyme (Sigma)] and lysed with an Avestin Emusiflex by passing the solution three times. Lysate was centrifuged for 30 min at 25,000 × g followed by an ultracentrifugation step of the supernatant for 1.5 h at 150,000 × g. The membrane pellets were resuspended in 2 ml buffer/g membrane pellets (30 mM Tris-HCl pH 7.5, 250 mM NaCl, 10% glycerol), flash frozen in liquid nitrogen, and stored at −20°C. Membranes were solubilized in 10 ml/g pellet buffer [30 mM Tris-HCl pH 7.5, 250 mM NaCl, 10% glycerol, and 1% n-Dodecyl-β-D-Maltoside (DDM, Glycon Biochemicals GmbH)] by stirring for 2 h at 4°C, followed by centrifugation at 50000 × g for 20 min. To the supernatant, 10 mM imidazole was added and applied to 2 × 3 ml ROTI®Garose-His/Ni NTA-Beads (Roth) in a gravity column. The column was washed with 20x column volume (CV) of buffer (30 mM Tris-HCl pH 7.5, 300 mM NaCl, 10% glycerol, 25 mM imidazole, 0.03% DDM) and eluted with 5 CV buffer with 250 mM imidazole. Elution fractions containing the protein were collected, and His-tag was removed by adding 3C-protease and an overnight incubation at 4°C. A “reverse” nickel chromatography was performed; the protein was concentrated with a 50 kDa MWCO Amicon Ultra 4 ml filter, followed by a size exclusion chromatography (SEC) with a Superdex 200 10/300 column; and here, the used buffer was DDM-buffer (30 mM Tris-HCl pH 7.5, 250 mM NaCl, 0.03% DDM) or LMNG-buffer (30 mM Tris-HCl pH 7.5, 250 mM NaCl, 0.001% lauryl maltose neopentyl glycol (LMNG, Anatrace)). For reconstitution of IJ1, amphipol A8-35 (Anatrace) was added in a 1:5 ratio (2 mg/ml protein: 10 mg/ml amphipol) to the protein in a total volume of 1 ml. 50 µL of a 70% slurry of buffer equilibrated and degassed SM-2 Bio-Beads (Bio-Rad Laboratories) was added to the mixture before incubation for 12 h at 4°C. After Bio-Bead removal, the sample was concentrated to 120 µL in 50 kDa MWCO Amicon Ultra-0.5 ml centrifugal filters and loaded onto a SEC column S200 Increase 3.2/300 or Superose 6 Increase 3.2/300 (30 mM Tris-HCl pH 7.5, 250 mM NaCl, 0.02% A8-35). Peak fractions containing IJ1 were pooled, aliquoted, flashfrozen with liquid nitrogen, and kept at −80°C until further use. Chromatograms are shown in the supplementary information (Supplementary Figure S1).

**TolC**: TolC protein was expressed in *E.coli* C43(DE3)ΔAcrAB cells. Cultures were grown in terrific broth (TB) media at 37°C and 200 RPM to an OD_600nm_ of 1.3, and expression was induced with 0.5 mM IPTG. Cultures kept growing overnight (∼16 h) at 20°C and 200 RPM. Cells were harvested by centrifugation (5,000 × g, 15 min, 4°C). Pellets were flashfrozen with liquid nitrogen and stored at −80°C until further use. To obtain the membrane fraction, cells were resuspended in 5 ml/g pellets in lysis buffer (20 mM Tris-HCl pH 7.5, 150 mM NaCl, 2 mM MgCl_2_, Protease Inhibitor (ROCHE), spatula DNaseI) and lysed with a LM10 microfluidizer (Microfluidics) by passing the solution four times at 10000 PSI. Cell debris was pelleted at 12000 RPM for 30 min at 4°C. Subsequently, membranes were pelleted by centrifugation at 35000 RPM for 1 h at 4°C. The pelleted membranes were resuspended in loading buffer (20 mM Tris-HCl pH 7.5, 150 mM NaCl, 10 mM ImidazoleHCl pH 7.5) at a concentration of 10 ml/g pellet. TolC protein was solubilized by adding DDM (Carl Roth, CN26) to a final concentration of 1% by stirring for 1 h at 4°C followed by a centrifugation at 35000 RPM for 1 h at 4°C. Cleared lysate was applied to a 5 ml Hitrap HP column using an automated ÄKTA system. After application, the column was washed with 15 CV loading buffer to which 0.03% DDM was added. To minimize contaminants, the column was washed with 10 CV washing buffer (20 mM Tris-HCl pH 7.5, 150 mM NaCl, 40 mM ImidazoleHCl pH 7.5, 0.03% DDM) and TolC was eluted using 10 CV elution buffer (20 mM Tris-HCl pH 7.5, 150 mM NaCl, 290 mM ImidazoleHCl pH 7.5, 0.03% DDM). Elution fractions containing the target protein were pooled and loaded onto a size exclusion chromatography column S200 16/600 PG equilibrated with SEC buffer (20 mM N-2-hydroxyethylpiperazine-N′-2-ethanesulfonic acid (HEPES)/NaOH pH 7.5, 150 mM NaCl, 0.03% DDM). Fractions containing trimeric TolC were pooled and concentrated using 50 kDa MWCO Vivaspin until 5 mg/ml concentration. Protein was aliquoted and flash frozen with liquid nitrogen and kept at −80°C until further use. Chromatograms are shown in the supplementary information (Supplementary Figure S2).

**ANTH and ENTH**: The expression and purification of human and yeast proteins are already described elsewhere (Garcia-Alai et al., 2018). Recombinant human ENTH domain from epsin-1 (hENTH) was expressed in *E. coli* BL21 DE3 (Novagen) as GST-fusion protein containing an N-terminal His-tag and a TEV (Hisx6-GST-TEV) cleavage site. 800 ml cultures in LB media were grown at 37°C shaking at 180 RPM until an optical density at 600 nm (OD600) of 0.8 was reached. After induction with 0.5 mM IPTG, the cultures were grown at 20°C for 4 h and harvested by centrifugation (4,000 × g for 30 min at 4°C). The cell pellet was lysed by sonication in the presence of 1 mg/ml DNase in 50 mM Tris-HCl pH 7.5, 250 mM NaCl, and 20 mM imidazole. Lysed cell extract was centrifuged (17,000 × g, 45 min at 4°C), and the supernatant was purified by nickel-nitrilotriacetic acid (Ni-NTA) purification (Qiagen). Protein was eluted in a final elution buffer of 50 mM Tris-HCl pH 8.0, 250 mM NaCl, and 250 mM imidazole. Excess of TEV protease was added to the imidazole-eluted fractions for cleavage of the Hisx6-GST tag. Digestion was performed during dialysis at 4°C overnight against 4 L of 50 mM Tris-HCl pH 8.0, 250 mM NaCl, and 1 mM dithiothreitol (DTT). To remove the tags, the dialyzed fractions were subjected to a second Ni-NTA, and the flow-through was concentrated to 5 mg/ml to be then injected in a size exclusion chromatography (SEC). SEC was performed using an ÄKTA liquid chromatography system (Amersham Biosciences) and a Superdex 75 10/300 GL (Tricorn) column (GE Healthcare) in 20 mM Tris-HCl pH 8.0 and 250 mM NaCl, 1 mM DTT. After SEC, the fractions were pooled and concentrated to 10 mg/ml and flash-frozen in liquid nitrogen and stored at −80°C. Chromatograms are shown in the supplementary information (Supplementary Figure S3).

**FIGURE 2:**
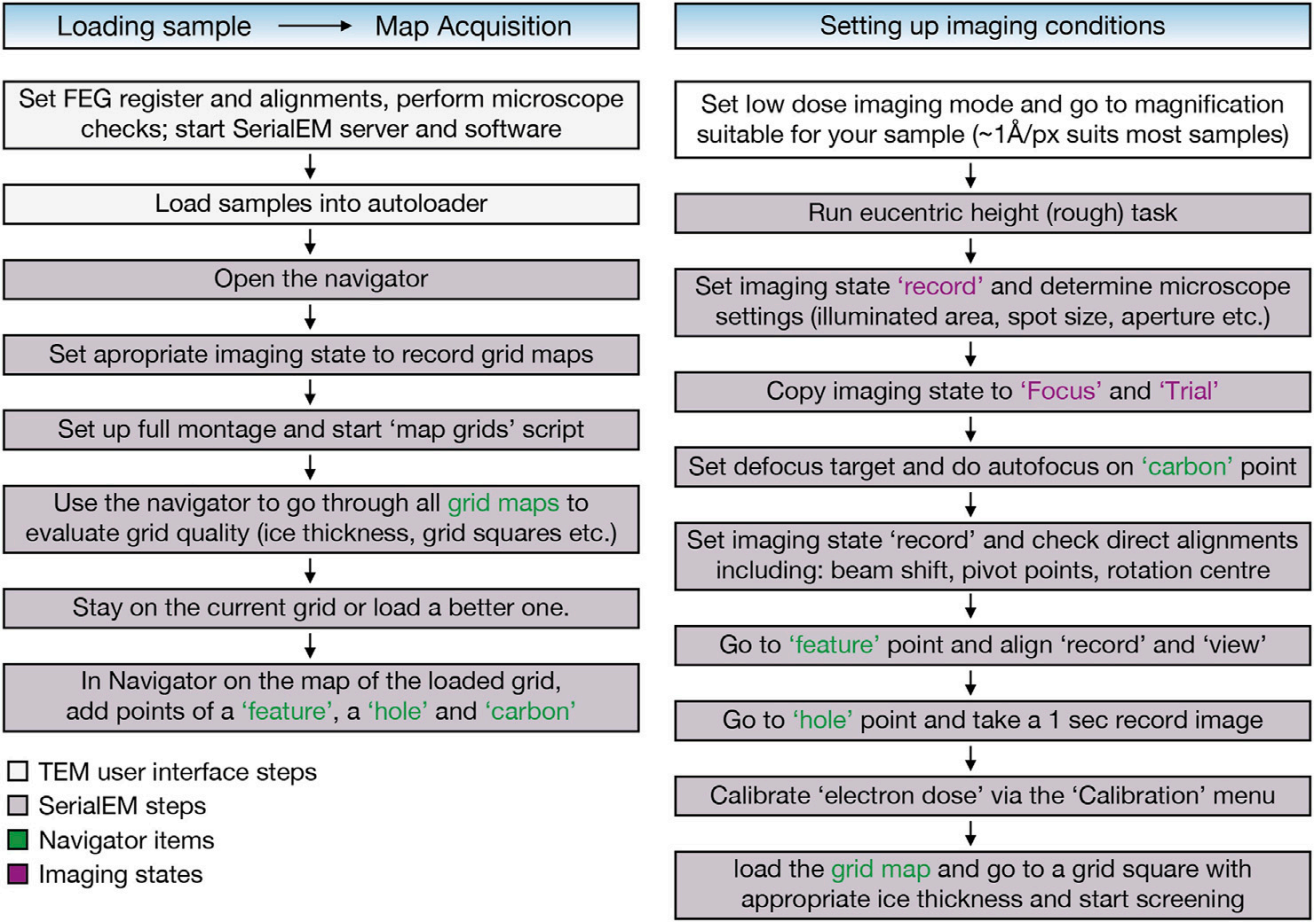
EM sample screening workflow using the SerialEM software (Mastronarde, 2005) includes automatic grid map acquisition of all loaded grids using the “map grids” script (https://serialemscripts.nexperion.net) followed by setting up imaging conditions for visual inspection of the samples.

**Electron microscopy and data collection**: Grids were screened according to the workflow depicted in Figure 2 on a Thermo Fisher Scientific 200 kV Talos Arctica equipped with a Falcon 3 EC direct detector or a 300 kV Titan Krios G3i equipped with a Gatan Bioquantum energy filter and K3 direct detector. Grid maps and images were recorded using SerialEM (Mastronarde, 2005) with scripts from the SerialEM Script Repository (https://serialemscripts.nexperion.net/). Negative Staining electron micrographs of TolC were acquired on a Jeol 200 kV cryo-TEM using in-house carbon coated grids. Data collection for IJ1 was done on the same Titan Krios system using EPU (Thermo Fisher Scientific). The electron dose rate was 15 electrons/pixel/s with a total dose of 60 electrons/Å^2^ (∼1 electron/A^2/frame). 3,228 movies were collected at 0.681 Å/px.

**Cryo-EM Data Processing**: Motion correction of movies was done using Relion 3.1.3’s own implementation (Zivanov et al., 2018). The contrast transfer function (CTF) was estimated using GCTF (Rohou and Grigorieff, 2015). Automated particle picking was performed in WARP (Tegunov and Cramer, 2019), coordinates for 417375 particles were imported in Relion 3.1.3, and 2D classification was performed after particle extraction (Zivanov et al., 2018).

**Mass photometry**: All measurements shown here were acquired on a commercial Refeyn OneMP mass photometer using the programs Acquire MP v2.5.0 and Discover MP v2.5.0 (Refeyn Ltd.).

## Stepwise Procedures

### Differential Scanning Fluorimetry (nDSF)

**Required materials**:

- Prometheus NT.48 Series nanoDSF Grade High Sensitivity Capillaries from Nanotemper
- Prometheus NT.48 from Nanotemper
- Buffers/detergents of interest
- Sample volume: usually around 50 µL for having several measurements (10 µL are needed for each measurement)

**Stepwise procedure**:

1. Having an initial idea of the extinction coefficient of the protein of interest can be helpful to determine the final experimental concentration. Check the amount of Trp and Tyr residues (and their position, if known) present on your protein, and usually aim for a final concentration between 5 and 10 µM. This concentration can be significantly lower for large proteins or proteins with a high number of Trp and Tyr residues.
2. Prepare a set of three serial dilutions from your stock of protein and do an initial scan with the Prometheus to observe the signal of the different dilutions. Also add the buffer of interest as control in order to rule out signal interference caused by buffer components.
3. Adjust the excitation power so that all of the samples are in the recommended regime of initial fluorescence (between 2000 and 15000 counts)
4. Perform a dilution of your protein in the buffers of interest using the same dilution performed for the initial scan to ensure a good initial signal for the experiment.
5. Remember to include a capillary with buffer only as a control to discard possible fluorescence effects coming from the buffer. In case of performing a titration with a compound (e.g., ligand), a buffer control with the highest concentration of the added compound must be measured in order to discard fluorescence contribution from the compound.
6. Load the nDSF capillaries, set a measurement from 20°C to 90°C using a heating rate of 1°C/min, and start the experiment.
7. While running or before: Add labels for each capillary in the acquisition software to know which sample corresponds to each curve.
8. Export the processed curves from the instrument and analyze the data using the SPC web server for the MoltenProt module (available at https://spc.embl-hamburg.de/).

### Dynamic Light Scattering

The following instructions are valid for a Wyatt DynaPro Nanostar. Other DLS machines can be used as well. However, the described stepwise procedure needs to be adapted individually for each instrument type.

**Required materials**:

- 4 µL Wyatt cuvettes
- Buffers of interest
- DynaPro Nanostar device (Wyatt Technology Corporation)
- Spin filters to remove large aggregates (e.g., Durapore^®^ Membrane Filter, 0.22 µm from Millipore^®^)
- Protein stock: 10 ul with a protein concentration of ca. 0.5 mg/ml

**Stepwise procedure**:

1. Measurements should be performed at a concentration of around 0.5 mg/ml in order to obtain a good signal (starting at a concentration of around 1–2 mg/ml is usually a good starting point). The volume required per measurement depends on the instrument and cuvettes used. In our case, we have used 4–5 µL of sample.
2. Before measurements, spin the samples at maximum speed for 10 min to avoid aggregates that could hamper measurements. Optionally, 0.22 μm mini-spin filters are recommended to be used to remove large aggregates from the sample.
3. To enhance the quality of the measurements, switch on the instrument and the laser at least 30 min before measuring to warm up the laser. Also, set the temperature (usually 20 or 25°C).
4. Measure the buffer to discard any signal that could come from buffer components such as detergent micelles. If the buffer contains impurities that display a particle-like auto-correlation function, it probably needs to be filtered.
5. Set the collection parameters to 30 curves and average the results. The acquisition time of 5 s with a total of 30 acquisitions averaged. Measurements to be performed at 25°C.
6. If the curves show a “bump” towards higher correlation-times, the sample contains large macromolecular aggregates and it is most likely not in ideal buffer conditions and therefore not suitable for structural studies (see Supplementary Figure S4A for an example).

#### Dynamic Light Scattering Data Analysis

The measured autocorrelation curve of the sample of interest can be analyzed with a variety of algorithms to obtain the hydrodynamic radius of gyration (Hr) (Koppel, 1972; Provencher, 1982; Schuck, 2000). This includes fitting one/two Hrs, a smooth distribution of Hrs, or a distribution of Hrs that follow a certain function (e.g., a Gaussian). The standard way of analyzing the autocorrelation function consists of employing the so-called method of cumulants (Koppel, 1972); however, this analysis can be completely hampered by really small amounts of aggregates. The other algorithms are more robust, but the estimated hydrodynamic radius should be nevertheless considered to be in all cases semi-quantitative. Regarding the DynaPro Nanostar device, results from fitting the data with the cumulants method and a smoothed distribution are provided.

### Mass Photometry

#### Cleaning of Cover Slides for Mass Photometry

The sonication is executed in batches of 10 microscope coverslips following the instruction of Soltermann et al. (2020).

**Required materials**:

- Microscope coverslips (CG15KH - Precision Cover Glasses from Thorlabs)
- Rack for microscope cover slips (e.g., from Electron Microscopy Sciences, catalogue number 72243)
- 600 ml beaker
- Ultrasound bath
- MilliQ Ultrapure water, e.g., Millipore^©^ system filtered water
- Isopropanol
- Nitrogen outlet for drying coverslips
- Container for storing coverslips (e.g., GlW, Slidebox K25W)

**Stepwise procedure**:

1. Place the microscope coverslips in a suitable rack (EMS, Adjustable Cover-Slip Rack, #72243).
2. Place the rack with the coverslips in a 600 ml beaker and fill this beaker with ultrapure water so that the slides are fully submerged.
3. Sonicate for 5 min at full power.
4. Remove water from the beaker and repeat steps 2.+3. with isopropanol.
5. Remove isopropanol and repeat steps 2.+3. with ultrapure water (optional: the isopropanol can be stored and reused).
6. Remove water and dry each coverslip under a stream of dry nitrogen.
7. Cleaned coverslips can be stored in a clean container (GlW, Slidebox K25W) with inserted spacers.

#### Buffer Filtering Prior to the Mass Photometry Experiment

**FIGURE 3:**
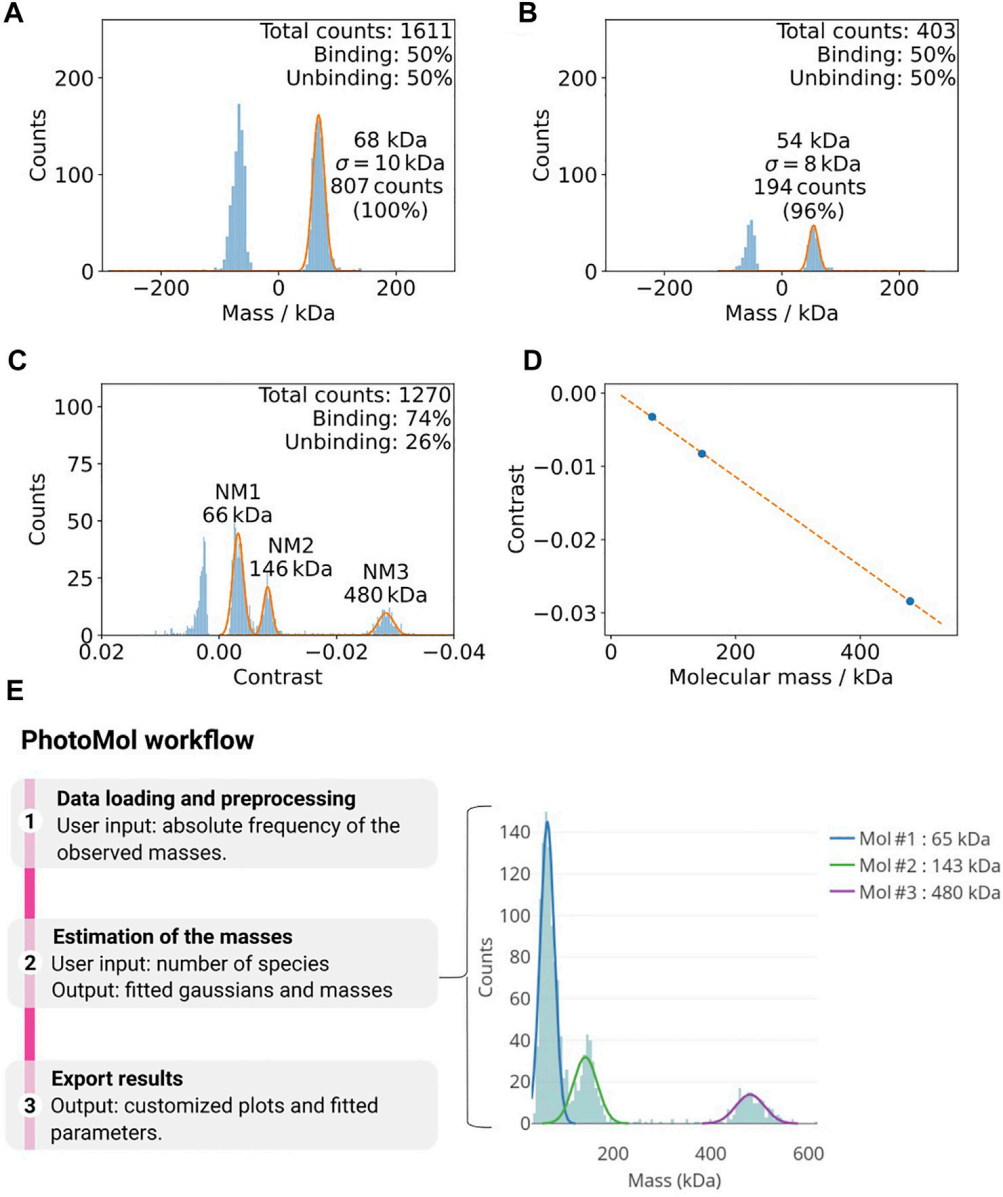
**(A,B)** Example of how buffer impurities can negatively affect mass photometry measurements (Tris-HCl 100 mM, pH 8.5, 100 mM NaCl, 1 mM DTT). **(A)** The vacuum filtered buffer on the left shows a considerable amount of counts at ca. 68 kDa. **(B)** An additional filter step (right) drastically decreases this background. **(C,D)** Calibration of mass photometer. **(C)** Histogram of the mass distributions used for the calibration of the mass photometer using the native marker in PBS buffer (pH 7.4). The Gaussian fittings for the different populations are shown in orange. **(D)** The contrast values from **(A)** and the known masses of the calibration standards are used for a calibration line (maximum error here 0.3%). **(E)**The PhotoMol pipeline consists of three steps. First, the user loads an input file that contains the frequency of the observed masses (or contrasts). Pre-processing is first performed by selecting a bin width and a window range to build the histogram. Second, the user defines the number of Gaussians (species) present in the distribution and a truncated multi-Gaussian fit is executed. Finally, publication-grade figures can be downloaded together with information about the fitted parameters.

Due to the high background signal of detergent containing buffers, it is often necessary to dilute the protein into detergent-free buffer prior to the mass photometry measurement. A key factor for successful mass photometry measurements is ensuring a low background signal from buffers during the measurement. It can occur that even detergent-free buffers used for other biophysical techniques yield a significant number of counts that can hamper accurate mass determination. In many cases, we could observe a strong signal in the region around 50–80 kDa (Figure 3A,B). In our experience, filtering with a 30 k spin filter (e.g., Amicon Ultra 30 k) could in these cases drastically decrease the background signal and enhance the data quality.

**Required materials**:

- 30 kDa spin filter (e.g., Amicon Ultra 30 k)
- Table-top centrifuge
- Detergent-free buffer

**Stepwise procedure**:

1. Fill the spin filter with buffer and centrifuge with the speed and time recommended for this spin filter.
2. Discard filtered solution.
3. Repeat step 1. and 2. (equilibration of filter device).
4. Repeat step 1. once more.
5. The filtered buffer is now ready to use.

#### Calibration of Mass Photometer

The mass photometer detects the signal in terms of contrast, which can be transformed into mass, thanks to a reference calibration using protein standards. The accurate detection of masses requires the diligent performance of this calibration. Usually, a calibration with the standard native marker uses three points to calibrate the mass with a maximum error that should be lower than 5% (Figure 3C,D).

**Required materials**:

- Protein marker (here: NM, Invitrogen, NativeMarker Unstained Protein Standard, #LC0725)
- Buffer that will later be used for the protein of interest
- Mass Photometer (here: Refeyn One)
- Computer with AcquireMP and DiscoverMP software
- Cleaned coverslip
- Reusable silicone gaskets (Sigma GBL103250-10 EA or Grace BioLabs 103250)
- Optional: Vortex for mixing protein standard dilution

**Stepwise Procedure**:

1. Add 98 μL of buffer to 2 μL of the native marker. Mix thoroughly by pipetting up and down or using a vortexer.
2. Add 18 μL of the buffer in an empty well on the coverslip.
3. Select a suitable field of view (FOV) depending on the mass of the protein of interest. For most systems, the regular FOV is suitable.
4. Focus the laser manually or using the autofocus function.
5. Add 2 μL of pre-diluted native marker (from 1.) and mix by pipetting up and down using a 20 μL pipette. Note: Only the proteins with masses corresponding to 66, 146, 480, and 1,048 kDa are visible with the instrument. The higher mass is however only detectable with the medium and high field of view (FOV). In our pipeline, we use the regular FOV where only 66, 146, and 480 kDa masses are detectable.
6. Start the acquisition as soon as the mixing is finished.
7. Open the mp file with DiscoverMP and analyze data.
8. Check if the number of counts is in the recommended range for the used FOV and the particular instrument type. For a RefeynOne and the regular FOV, the recommended maximum number of counts is 3000. Note: Ideally, the majority of counts should be attributed to binding events. However, for different buffer systems, the ratio between binding and unbinding events can differ.
9. If the number of counts is too high or too low, repeat steps 4.–8. adjusting the dilution of the native marker.
10. Once the number of counts is satisfactory, fit Gaussians to the peaks. For the regular FOV, we usually use the peaks of bovine serum albumin (NM1: 66 kDa), lactate dehydrogenase (NM2: 146 kDa), and apoferritin band 2 (NM3: 480 kDa). For medium and large FOV, we usually use NM2, NM3, and NM4 (1,048 kDa).
11. Calibrate with the calibration function in the software and save the calibration file. This can be used for calibrating the measurement of the sample of interest later.
12. Process the raw data using DiscoverMP and export the events fitted file (h5 format) for later use with the webserver.

#### Mass Photometry of Protein of Interest

**Required materials**:

- Filtered buffer
- Mass Photometer (here: Refeyn One)
- Computer with the AcquireMP and DiscoverMP software
- Cleaned coverslip
- Reusable silicon gaskets (Sigma GBL103250-10 EA or Grace BioLabs 103250)
- Protein stock: 2–10 μl at a concentration of at least 500 nM

**Stepwise procedure**:

1. Create a pre-dilution of the protein stock with a protein concentration of 500–1,000 nM using filtered buffer.

Note: For concentrated detergent samples (those proceeding from concentrating devices), we recommend starting with a pre-dilution with protein concentration around 1.5 μM and then using only 0.5 μL of it (and 19.5 μL of non-detergent buffer on the slide) in step 3. This could highly reduce the detergent background in the cases presented here.

2. Add 18 μL of filtered detergent-free buffer into an empty well on the cover slide.
3. Add 2 μL of pre-diluted protein solution (from 1.) and mix by pipetting up and down using a 20 μL pipette.
4. Start the acquisition as soon as the mixing is finished.
5. Open the mp file with DiscoverMP and analyze data.
6. Check if the number of counts is in the recommended range for the used FOV (this depends also whether a RefeynOne or RefeynTwo is used). For a RefeynOne and the regular FOV, the recommended maximum number of counts is 3000.
7. If the number of counts is too high, repeat steps 2–6 using a smaller volume of the pre-diluted protein solution in a new well.

#### Important Quality Checks for Mass Photometry

- The cleanliness of the slide can be checked after adding the buffer. Local impurities show up as bright spots in the native view. Move the objective position by changing the x/y position of the stage with the Acquire MP software. If no clean area can be found in a well, change to another well or use another slide.
- Always check the signal of the pure buffer before measuring the protein of interest. If a high number of counts (larger than a few 100) is detected, this could be caused by the detergent. In this case, try to decrease the detergent concentration by diluting it below its CMC. This is a requirement for MP measurements since concentrated detergents lead to high levels of noise background. Given that the measurement is fast, the membrane protein mass remains adequate during the measurement (Olerinyova et al., 2020). For instance, the mass photometry with TolC shown later (cf. Figure 7) was acquired with one third of the CMC of DDM. For other detergents, this needs to be empirically confirmed case to case. A good summary of different detergents and their respective signals at different concentrations can be found in this application note by Refeyn: https://www.refeyn.com/mass-photometry-with-detergents.

#### Mass Photometry Data Analysis

Mass photometry data acquired on a Refeyn instrument can be analyzed using the proprietary software that requires a license by Refeyn DiscoverMP. Here, we are releasing a new user-friendly software module for MP analysis and publicly available at the eSPC data analysis platform: spc.embl-hamburg.de (Burastero et al., 2021). This is particularly important when the proprietary software is not accessible. It allows fast and easy high-quality data analysis with the possibility of exporting publication-grade figures (Figure 3E). The eSPC module PhotoMol allows quantifying the masses of different species in a sample after a mass photometry experiment. The required input file is an .h5 file (data file saved in the hierarchical data format) with the fitted events generated by the software Refeyn DiscoverMP. In the DiscoverMP version <2.5, the file events, Fitted.h5 is saved in the folder when saving the results. In version 2.5, the events can be exported individually selecting a custom file name. Moreover, a comma-separated-values (csv) file can be also loaded. If the file was generated after mass calibration, the masses in kDa are included. In case that only the contrasts are present (“contrasts” dataset in the .h5 file), another file with known masses can be used for calibration and to transform the observed contrasts into masses.

Once the one-dimensional dataset of the observed masses is loaded, a histogram is built based on a chosen bin width and range. The estimation of the masses consists of fitting a truncated multi-Gaussian to correctly fit data with multiple mass distributions. An initial number of Gaussians based on detected peaks are provided but should be only used as a starting point. The user should change, if desired, the number of Gaussians together with initial guesses. We chose to fit left-side truncated Gaussians to take into account the mass range of the instrument, i.e., there are no counts for masses below a certain threshold (i.e., below 30 kDa). Further information regarding the PhotoMol software is available as Supplementary Information (PhotoMol User Documentation).

### Sample Preparation for Negative-Stain EM

**Required materials**:

- Protein Stock Solution: 20 μl at a concentration of 2–20 μM for serial dilutions
- Protein Buffer (low salt, low phosphate). Note: In our experience, salt concentrations higher than 300 mM and phosphate containing buffers should be avoided as this may lead to precipitations on the grid.
- Carbon or Carbon/Formvar support film on copper grids 300 mesh
- 2% uranyl acetate solution
- Harrick Plasma Cleaner PDC-002-CE, GloQube or similar instrument
- High quality tweezers like Dumont Type N5 for EM grids (from Plano)
- Whatman filter paper grade 1
- Designated bench space and waste container to handle uranyl acetate samples

Hazards: Uranyl acetate is radioactive and toxic.

**Stepwise procedure**:

Note: Negative staining is not necessarily the best method to characterize membrane proteins. It has been reported (Boekema, 1991) that the heavy metal stain, in combination with the lipid/detergent environment can cause aggregation. However, it is quick and easy to do, and may allow a quick first assessment of sample quality.

1. An EM specimen is typically prepared using 3.5 μl protein solution at a concentration of 0.05–5 μM. Set 20 μl of your stock solution aside.
2. Glow-discharge carbon-coated grids with the glow discharger for 60 s using negative polarity at 25 mA intensity. Make sure to use freshly glow discharged grids as the glow discharging effect degrades over time.
3. Prepare a few (between 10–100 fold) dilutions of your protein stock solution in its buffer based on the previous characterization, preferably using low salt and no phosphate.
4. Attach a slice of parafilm with a few drops of water on a surface designated to work with heavy metals.
5. On the parafilm, prepare for each sample a row of droplets starting with two 20 μl droplets of MilliQ water and two 4 μl droplets of a 2% uranyl acetate solution.
6. Using the tweezers, grab a glow-discharged grid only on the copper rim at the edge.
7. Apply 3.5 μl of a sample on top of the glow-discharged grid without touching the grid with the tip of the pipette.
8. Incubate the sample on the grid for 30 s.
9. Carefully remove excess liquid by side-blotting the grid. Proceed before drying the grid completely.
10. Quickly dip the grid into the first water droplet to remove unbound proteins. Then remove the excess water by side blotting. Repeat with the second droplet of water. Proceed with staining before drying the grid.
11. Quickly dip the grid into the uranyl acetate droplet and blot. Then incubate on the second uranyl acetate drop for 1 min. Carefully remove excess stain by side blotting.

Note: Uranyl acetate is a hazardous substance. Alternatives are ammonium molybdate and phosphotungsten acid, NanoVan & NanoW (Tedpella Inc.). However, in our experience, these alternatives do not result in such a good contrast as the one obtained with uranyl acetate.

12. Finally, let the grid air-dry for 2 min. Store in a grid box until the sample can be imaged.

### Cryo-EM Sample Preparation for Single Particle Analysis of Membrane Proteins (Preferred)

Vitrification of membrane proteins in different buffers and/or detergents is the best method to assess the sample behavior. In most cases, sample morphology, protein homogeneity, and dispersity of particles on EM grids including aggregation and particle concentration can only be determined from vitrified EM specimens. In our experience, membrane proteins are notoriously difficult to be deposited into the holes of the grid during grid preparation and tend to localize on the carbon support. To increase the density of membrane proteins in grid holes, it is necessary to vary the used concentration, but also the type of grid support can have a substantial impact on protein distribution (cf. Figure 6). The following stepwise procedure can be used to prepare grids of any type, but the necessary conditions and especially the protein concentration–grid type relation have to be determined individually. Our standard starting setup uses QuantiFoil MultiA Cu 200 grids and three dilutions of the protein of interest (e.g., 1, 0.3, 0.1 mg/ml).

**Required materials**:

- EM grids:
  ○ Copper grids 2 nm Carbon R2/1 Cu 200
  ○ Copper grids QuantiFoil R 1.2/1.3 Cu 200
  ○ Copper grids QuantiFoil MultiA Cu 200
  ○ Gold grids UltrAuFoil^®^ R1.2/R1.3 Au 300
- Blotting device like Thermo Fisher Vitrobot Mark IV
- Harrick Plasma Cleaner PDC-002-CE, GloQube or similar instrument
- Buffers/detergents of interest
- Sample volume: 20 µl of 20–200 µM is usually a good starting concentration.

**Stepwise procedure**:

1. Prepare a set of three dilutions from your stock of protein as 1:2–1:10 in the appropriate buffer. Usually, around 20 µl per dilution is needed for preparing several grids (3.5 µL is needed for each grid).
2. Fill cryoplunger reservoir with a maximum of 60 ml fresh deionized water using a syringe.
3. Equilibrate Thermo Fisher Vitrobot Mark IV to 4°C and 95% relative humidity in the climate chamber. Under “miscellaneous,” select the “switch off during process” option to avoid ice contamination due to the humidifier.
4. Assemble the dedicated styrofoam plunging container, the inner brass cup and the temperature conductor (spider), and the grid box holder. Cool down the plunging container with dry liquid nitrogen.
5. When the inner brass cup reaches liquid nitrogen temperature, slowly fill it with either ethane or a mixture of ethane and propane (63%/37%). The latter is preferably used as it does not freeze over time.
6. Remove the spider. Top-up the plunging container with liquid nitrogen, avoiding liquid nitrogen penetration into the brass cup.
7. Using grid box tweezers, put an empty grid box in the designated position and remove the lid.
8. Move to plasma cleaner, e.g., GloQube or Harrick and glow discharge grids. For Quantifoil MultiA grids, use 60 s at 25 mA (giving ∼350 V). For grids coated with a thin (2 nm) carbon film, reduce the glow discharge time to 10–20 s.
9. Move with freshly glow-discharged grids back to Vitrobot. Note: Use grids within the next 30 min, while the surface of the grids is hydrophilic.
10. Select the “place new grid” function on the display.
11. Pick up the first grid using a pair of dedicated Vitrobot tweezers and attach them carefully to the metal rod of the Vitrobot.
12. Select “continue” to transfer the tweezers into the climate chamber followed by “start process”.
13. Apply 3.5 µL of your sample to the grid using the side entry port.
14. Select “continue” to start the blotting and plunging procedure.

Note: The blot force is usually calibrated by service using millimeter paper. Changing the blotting force and time can have an influence on the resulting ice thickness and ice gradient on the grid. It makes sense to start with generally used/“known to work” standard settings (wait time: 4 s, blotting force: 4, blotting time: 4 s in our case). After screening the first set of grids, these settings can be adjusted and tested. For the test protein used here, “Case 1—IJ1” blotting force settings between 0 and 4 and blotting times between 3 and 6 s were used.

15. After plunging the grid into the liquid ethane:propane container, move to grid transfer position.
16. Carefully detach the tweezers from the Vitrobot while keeping the grid in the liquid ethane:propane mix. Transfer the grid to the liquid nitrogen storage ring and then into the grid box for storage. During transfer keep the grid in either liquid or gas phase nitrogen to minimize ice contamination.
17. After the grid has been stored in its box, refill the liquid nitrogen storage ring with dry liquid nitrogen.
18. Pick up the next grid with the tweezers and continue as before starting with step 10 to prepare more samples. It is advisable to make duplicates of each sample.
19. After the last grid is prepared, you may either continue (a) imaging grids, (b) clipping grids, and (c) transfer grid boxes for later use into a long-term liquid nitrogen storage Dewar.
20. Shut down and switch off the Vitrobot, and remove and empty the humidifier. Last, place the coolant container under a fume hood to allow evaporation of the remaining liquid nitrogen, ethane, and propane.

### EM Sample Screening

The EM sample screening protocol is depicted in Figure 2.

### Anticipated Results: Membrane Protein Examples

We applied the biophysics pipeline described above to three membrane protein systems.

#### Case 1: Integral Membrane Protein Ij1

**FIGURE 4:**
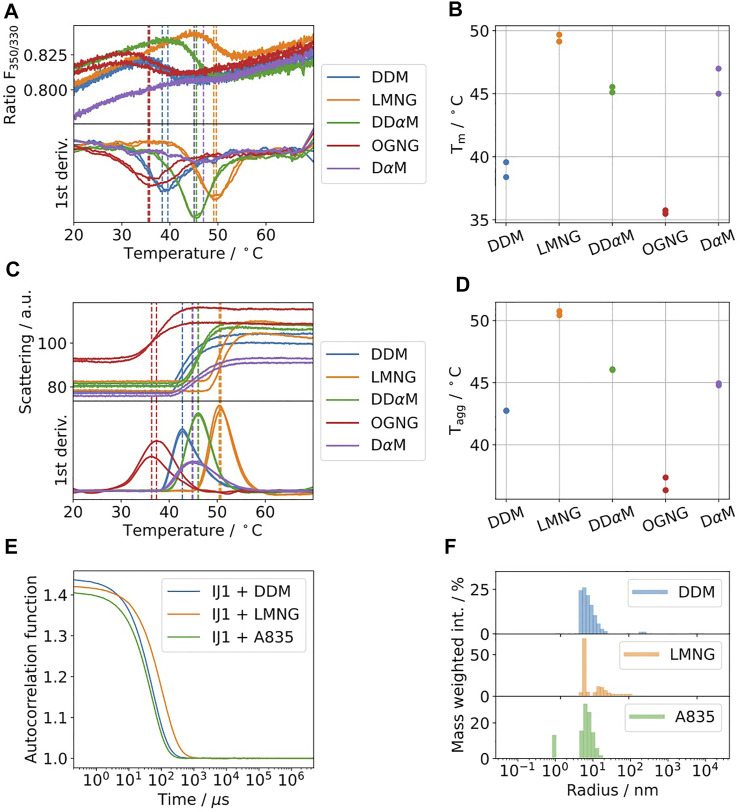
**(A–D)** Five commonly used detergents were selected for protein solubilization of IJ1. DSF fluorescence ratio of IJ1 for the selected detergents **(A)** and melting temperatures as determined by the minimum of the first derivative **(B)**. The backscattering signal **(C)** indicates aggregation and shows similar transition temperatures **(D)** as the fluorescence ratio. LMNG was identified as the detergent with the highest stabilization amongst a screen of 96 conditions. The detergent concentrations used here were 0.6 mM (0.03%) DDM (used as reference), 1 mM LMNG, 8.5 mM DD*α*M, 3.1 mM OGNG, and 4.8 mM D*α*M. **(E)** DLS autocorrelation function of IJ1 in the presence of 0.6 mM (0.03%) DDM and 0.5 mM (0.05%) LMNG and reconstituted in amphipols (A8-35). **(F)** Mass weighted intensity histograms for the autocorrelation functions shown in **(A)** obtained by the application of the Stokes–Einstein equation for determining the average size particle: DDM 11.0 nm (921.0 kDa), LMNG 5 nm (147.6 kDa) and 21.5 nm (4,394.2 kDa), and A8-35 0.9 nm (2.8 kDa) and 9.7 nm (683.1 kDa).

Ij1 is an *E.coli* ABC-transporter involved in ion transport (Kotov et al., 2019). To determine the optimal buffer condition for protein stability, differential scanning fluorimetry was used as described in Kotov et al. Sci. Reports 2019. Five different detergents were selected and are shown in Figures 4A–D. The highest melting temperatures were detected for LMNG, which are almost 10°C higher than the initial detergent used for membrane solubilization, DDM. The effects on the aggregation behavior were afterwards tested using DLS for samples solubilized in DDM and later exchanged to LMNG and amphipol A8-35 (Figure 4E,F). DLS data indicate similar distributions for the hydrodynamic radii for DDM and A8-35 with main radii of 11 and 9.7 nm, respectively. LMNG showed two separate distributions with hydrodynamic radii of 5 nm (72% of mass) and 21.5 nm (27% of mass). All three samples have good quality autocorrelation functions displaying only one inflection point and are considered to be monodisperse.

**FIGURE 5:**
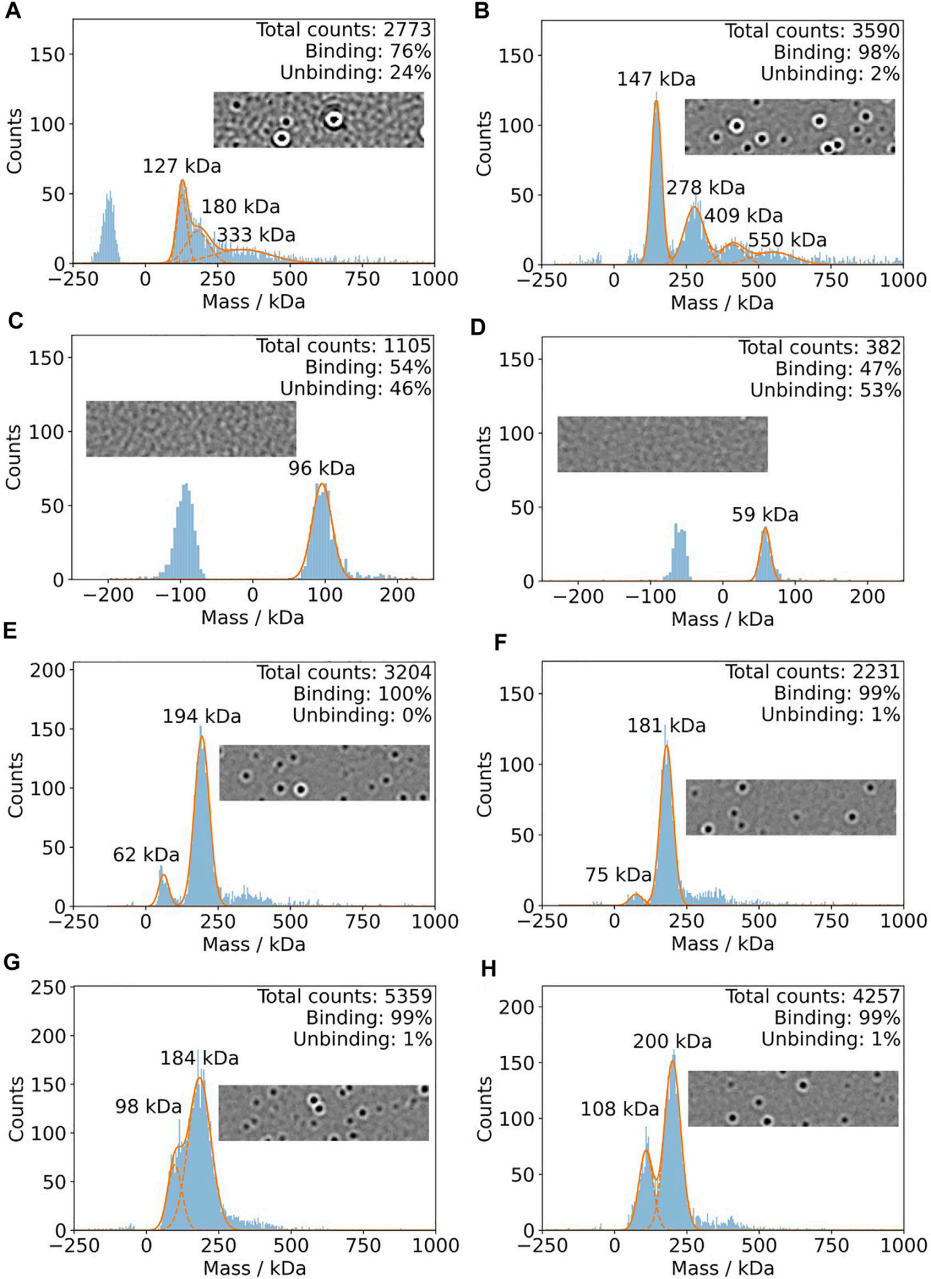
Mass photometry of IJ1. For each experiment, 0.5 ul of pre-dilution was added to 19.5 detergent-free buffer on the mass photometry slide. **(A)** Measurement using a protein pre-dilution with 0.036% DDM. The final DDM concentration is 0.0009% and 40 nM protein. **(B)** Pre-dilution of protein solution into detergent-free buffer results in a DDM concentration of 0.006%. The final DDM concentration is 0.00015% and 80 nM protein. **(C,D)** Control experiments using similar DDM concentrations and no protein. **(C)** 0.03% DDM. **(D)** 0.003% DDM. **(E)** and **(F)** show the mass histograms for IJ1 in the presence of different concentrations of LMNG as detergent at a final protein concentration of 40 nM. Since the protein was concentrated, we can only give estimations for the final LMNG concentrations: 0.00004% for panel E and 0.000015% for panels **(F)**, **(G)**, and **(H)**. Amphipol solubilized IJ1 at two different pre-dilution concentrations: **(G)** 3.2 uM IJ1 and 0.0042% A8-35. **(H)** 1.6 uM IJ1 and 0.0021% A8-35. The final protein concentrations were 80 nM **(G)** and 40 nM **(H)**.

In Figure 5, mass photometry experiments of IJ1 with LMNG, DDM, and A8-35 are shown. Mixing 0.5 µL of protein pre-dilution with a DDM concentration of 0.036% with 19.5 µL of detergent-free buffer on the slide resulted in broader mass distributions (Figure 5A) and a considerable amount of unbinding events, which did not allow reliable mass quantification. This became more obvious when looking at the oscillating background of the native image during the mass photometry measurement (inlay of Figure 5A). However, starting from a pre-diluted sample (0.006% DDM) in non-detergent buffer resulted in a more distinct mass distribution with clear peaks at 147 and 278 kDa (Figure 5B) and a very low number of unbinding events. The final DDM concentration for this measurement was around 0.00015%. The control measurements with buffer only for the two DDM concentrations are shown in Figure 5C,D. The background effect of the detergent LMNG is less pronounced compared to DDM. Even at the higher concentration of LMNG, the number of detected events is only slightly above 3000 counts (Figure 5E,F). When using amphipol A8-35 reconstituted protein, decreasing the protein concentration resulted in better separated peaks at 108 and 200 kDa (Figure 5G,H). In summary, LMNG and A8-35 solubilized IJ1 showed superior behavior compared to DDM.

**FIGURE 6:**
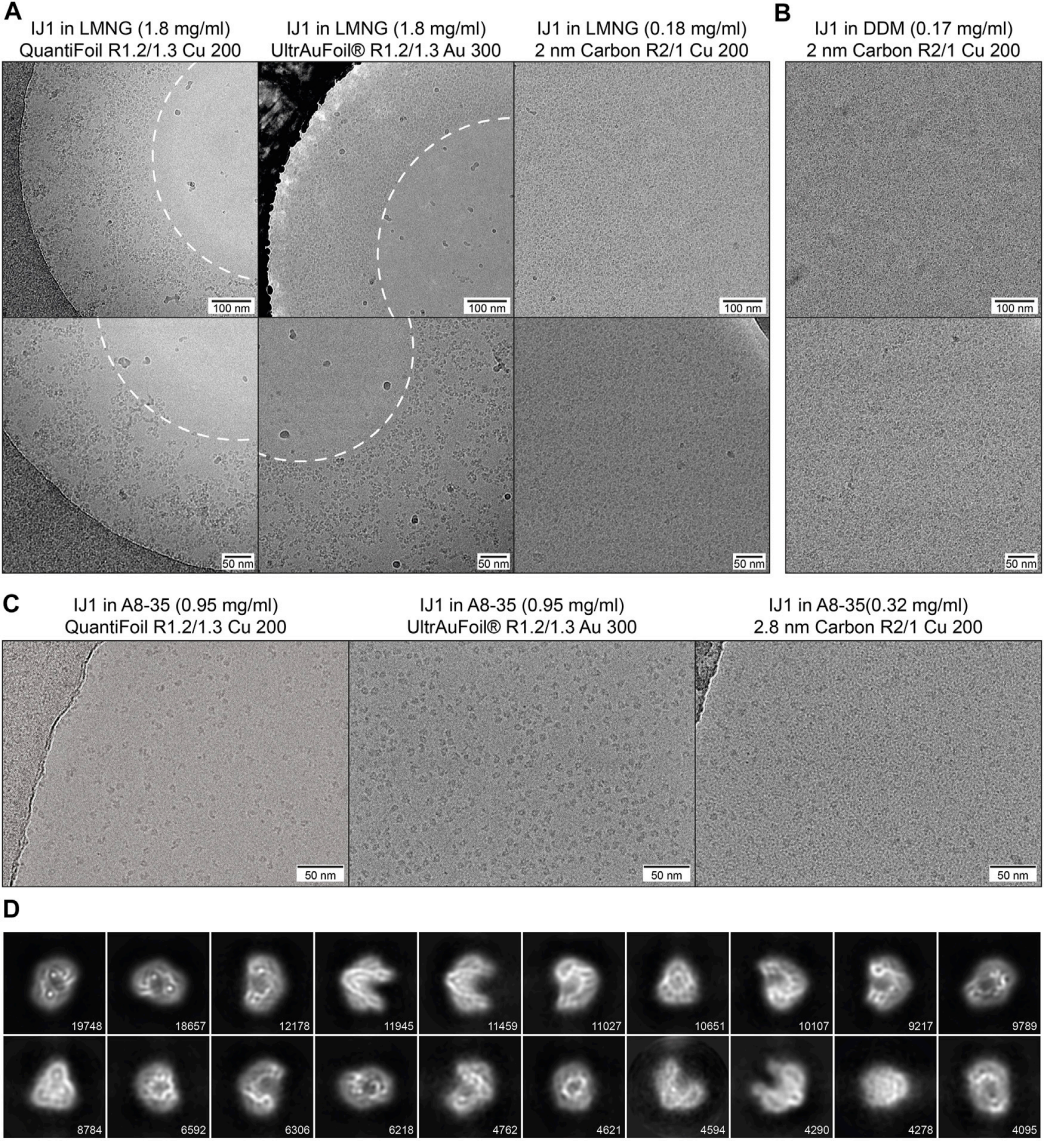
**(A)** Influence of different grid types on the particle distribution of IJ1 in LMNG. The protein localizes to regions of thick ice on copper QuantiFoil and UltrAuFoil grids and is omitted from regions of thin ice in the middle of the holes (marked by the white dashed line). A 2 nm layer of carbon leads to an even distribution of particles throughout the grid holes. **(B)** A similar distribution is seen for IJ1 in DDM on 2 nm carbon-coated grids. Shown are example images in two magnifications (1.58 Å/px upper row, 1.23 Å/px lower row). **(C)** IJ1 in A8-35 on QuantiFoil, UltrAuFoil, and 2.8 nm carbon-coated grids (0.68 Å/px). IJ1 reconstituted in A8-35 is more evenly distributed compared to LMNG solubilized particles. **(D)** 2D class averages for A8-35 reconstituted Ij1 on the QuantiFoil R1.2/1.3 grid. Shown are the 20 most populated classes of 80. The number of particles is shown for each class.

Three different detergent conditions were used for cryo-EM sample preparation of IJ1and combined with different grid types. This illustrates the effect of detergents and amphipol on the protein distribution and the influence of different grid supports on the protein distribution and density. On copper QuantiFoil and gold UltrAuFoil grids, IJ1 in LMNG localizes to regions of thicker ice and is omitted from regions of thin ice in the middle of the holes. Using grids with a 2 nm layer of carbon leads to an even distribution of particles throughout the grid holes (Figure 6A). The same effect is seen for the protein solubilized in DDM (Figure 6B). It should be noted that the 2 nm carbon layer leads to a slightly reduced contrast in the images and that protein concentrations 10 times lower than on holey grids should be used. Exchanging the detergent for amphipol A8-35 allows the protein to be more evenly distributed on holey grids without an additional carbon layer (Figure 6C). Particles in LMNG are more crammed together and often overlap each other, while A8-35 reconstituted particles are better separated. The improved display of amphipol reconstituted Ij1 on grids matches the biophysical results (Figure 5G,H, Figure 6C), resulting in well-aligned 2D class averages (Figure 6D).

#### Case 2: Integral Membrane Protein TolC

**FIGURE 7:**
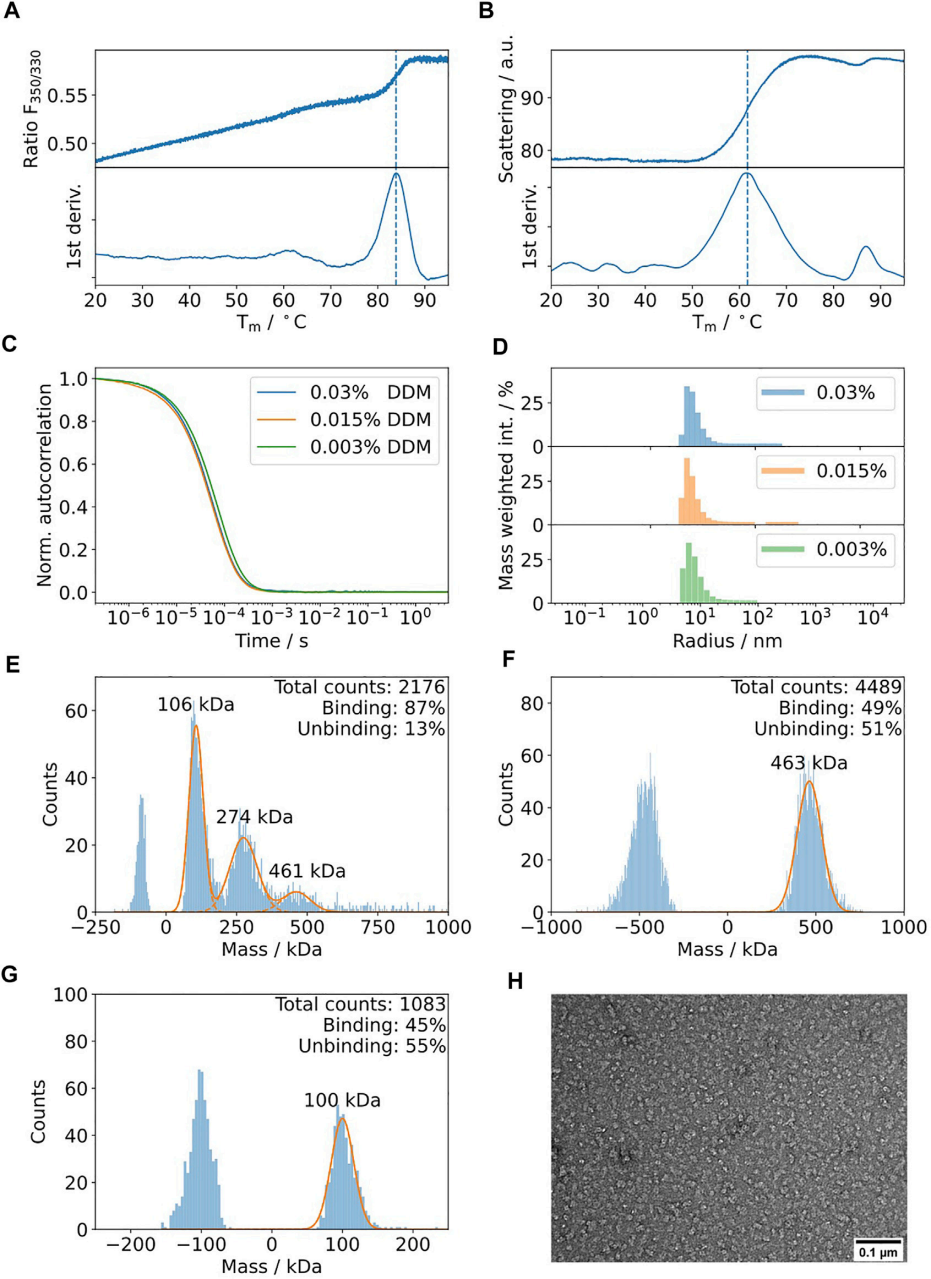
Biophysical characterization of TolC in detergent DDM. **(A)** nDSF of 5 μM TolC in 0.03% DDM buffer with a heating rate of 1°C/min indicates a high thermal stability with a weak transition around 84°C. **(B)** The scattering curve acquired during the nDSF indicates aggregation at a lower temperature of 62°C. **(C)** The DLS autocorrelation curves suggest similar polydispersity and hydrodynamic radii for DDM concentrations in the range of 0.003 and 0.03%. **(D)** Mass weighted histograms for the curves displayed in Figure 7C **(E)**. Mass photometry of 150 nM TolC in the presence of 0.003% DDM. Three bands are detectable at 106, 274, and 461 kDa. Control measurements at 0.03% **(F)** and 0.003% DDM **(G)** suggest that the bands at 106 and 461 kDa can be assigned to DDM. The band at 274 kDa can be assigned to the TolC-DDM complex (the theoretical mass of TolC is 162 kDa). **(H)** Negative staining electron micrographs of TolC at 0.015 mg/ml using 40000 x magnification on a Jeol 200 kV cryo-TEM using in-house carbon-coated grids.

TolC is an integral membrane protein from *Escherichia coli* (Husain et al., 2004) with a molecular weight of 161.7 kDa for its trimeric state. Collectively, the trimer forms a β-barrel that is embedded into the outer membrane and its α-helical part spanning into the periplasm. Upon assembly with AcrAB, its main function is the efflux of diverse molecules, such as toxins and antibacterial drugs (Koronakis et al., 2004). The results of the biophysics pipeline are shown in Figure 7. According to nDSF, the protein is highly stable in DDM with a dominant transition at 84°C (Figure 7A). Therefore, no further detergent screening was performed. It is important to note that this is based on the signal of one single tryptophan in the protein, probably buried in a more stable region of the protein (T_m_ of 84°C vs. T_agg_ of 62°C). Hence, for the identification of stabilizing conditions during sample preparation, it is relevant to consider the onset of scattering that relates to the change in the slope of the curve, equivalent to the temperature where 1% of protein aggregates (Figure 7B). Additionally, DLS was used to determine the lowest DDM detergent concentration that can be used to prevent the aggregation of the protein sample (Figure 7C,D). The results show that decreasing the DDM protein from 0.03 to 0.003% does not modify the autocorrelation curve. Therefore, mass photometry was performed at a concentration of 0.003% DDM, resulting in the detection of three peaks at 106, 274 and 461 kDa (Figure 7E). Based on control experiments at two different DDM concentrations (Figure 7F,G), the peaks at 106 and 461 kDa can be assigned to DDM empty micelles and the peak at 274 kDa can be assigned to DDM micelles with integrated TolC.

#### Case 3: Membrane Remodelling Complexes: hENTH and AENTH

**FIGURE 8:**
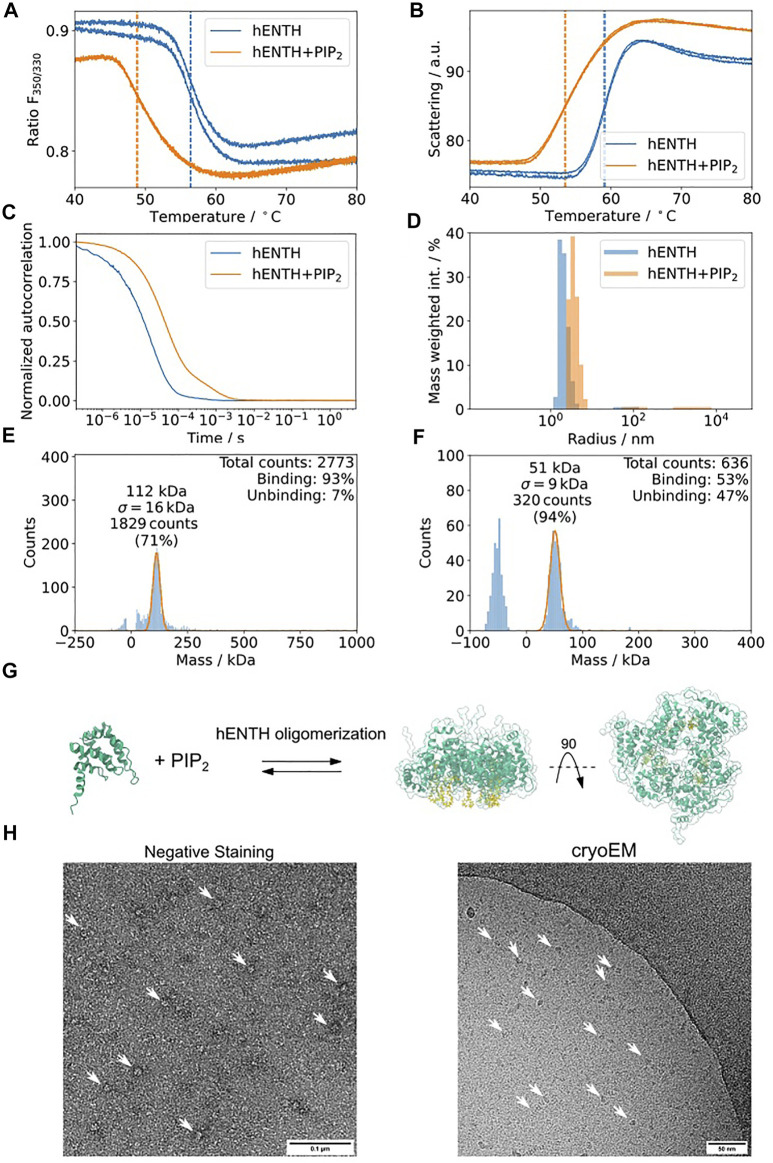
hENTH forms complexes in micellar concentrations of PI(4,5)P_2_. **(A)** nDSF transitions for hENTH at 30 µM in the absence and presence of 200 µM PI(4,5)P_2_. The shift in the sample with PI(4,5)P_2_ indicates that the oligomer unfolds before the monomers. **(B)** Scattering curves of hENTH in the absence and presence of 200 µM PI(4,5)P_2_. The hENTH aggregates at lower temperature in the presence of PI(4,5)P_2_. **(C)** DLS auto-correlation curves of hENTH at 30 µM in the absence and presence of 200 µM PI(4,5)P_2_. The shift in the autocorrelation curve indicates that the hENTH domain oligomerizes in the presence of PI(4,5)P_2_. **(D)** Histogram of the masses present on the DLS samples in the absence and presence of PI(4,5)P_2_. hENTH shifts towards higher masses in the presence of PI(4,5)P_2_, consistent with oligomer formation. **(E)** hENTH at 100 nM (monomer MW = 18.5 kDa) shows a peak at 108 kDa, corresponding to a hexamer of hENTH in the presence of 200 µM PI(4,5)P_2_ at 112 kDa. **(F)** Control: mass photometry histogram of buffer with 200 µM PI(4,5)P_2_. A peak at ca. 51 kDa corresponds to PI(4,5)P_2_ micelles. **(G)** Schematic of the hENTH domain (green) (PDB ID: 5ONF) and the oligomer model with PI(4,5)P_2_ molecules (yellow) from Garcia-Alai et al., 2018. **(H)** Representative negative staining and cryo-electron micrograph of hENTH + PI(4,5)P_2_ where hENTH hexamers can be observed. Particles of interest are indicated with white arrows.

Epsin is an adaptor protein involved in clathrin-mediated endocytosis (Ford et al., 2002; Yoon et al., 2010; Lai et al., 2012; Skruzny et al., 2015; Joseph et al., 2020). It contains an amphitropic membrane binding domain, hENTH (Epsin-N-terminal Homology), which induces membrane tubulation upon binding to the phospholipid PI(4,5)P_2_ (Ford et al., 2002). To structurally characterize these oligomers, we performed the proposed biophysical characterization of the sample using Dynamic Light Scattering (DLS), nDSF, and mass photometry (MP) of hENTH in the absence and presence of PI(4,5)P_2_ (Figure 8). hENTH shows a shift melting temperature (T_m_) in nDSF in the presence of PI(4,5)P_2_, indicating that the protein unfolds earlier when in the presence of the lipids (Figure 8A). The sample is also more prone to aggregation, as evidenced by the earlier T_agg_ from the static scattering measured as well using the nDSF device (Figure 8B). DLS experiments showed a shift in the auto-correlation curve when in the presence of 200 µM PI(4,5)P_2_ (Figure 8C). Plotting the radius of the particles in solution when in the presence of PI(4,5)P_2_ reveals a shift towards larger radius, indicating the formation of a soluble oligomer of hENTH domains (Figure 8D). Mass photometry revealed that a buffer containing 200 µM PI(4,5)P_2_ gives a distribution around 50 kDa, corresponding to the PI(4,5)P_2_ micelles present in the buffer (Figure 8F). While the mass of the monomeric hENTH (18 kDa) is not detectable by mass photometry, a clear mass distribution corresponding to 108 kDa and in agreement with what was previously described as an hENTH hexamer by SAXS and native MS (Figure 8G) is detected in the presence of 200 µM PI(4,5)P_2_ (Figure 8E). This characterization pipeline confirms that the particles observed in negative staining and cryo-EM micrographs of hENTH in the presence of PI(4,5)P_2_ correspond indeed to hENTH hexamers (Figure 8H).

Both DLS and MP can provide useful information regarding sample quality prior to cryo-EM sample preparation. To showcase the complementarity of both methods over a challenging sample, we have used membrane binding domains from endocytic adaptors Sla2 and Ent1 from yeast. During clathrin-mediated endocytosis, these proteins form a phosphatidylinositol 4,5-bisphosphate (PIP_2_)-dependent complex, essential for membrane remodelling and invagination. It has been shown that their membrane binding domains, ANTH and ENTH, oligomerize *in-vitro* into different assemblies through lipid interfaces forming the AENTH complex (Garcia-Alai et al., 2018; Lizarrondo et al., 2021). Here, DLS has been a powerful tool to assess the aggregation of the individual ANTH and ENTH domains upon mixing with PIP_2_ (Supplementary Figure S4A) and during the AENTH complex formation. However, even when DLS could help in the optimization of a non-aggregated sample, it would only provide an average radius of gyration of particles in solution (Supplementary Figure S4B,C). In addition, MP allowed us to accurately determine several of the AENTH assemblies providing a good platform for screening conditions that allowed us to determine the structure of the complexes by native mass spectrometry and single particle cryo-EM (Supplementary Figure S4D). Importantly, the macromolecular complex species identified as 12mers and 16mers by MP cannot be resolved by DLS (see the orange and red lines between 6 and 10 nm on the right panel of Supplementary Figure S4C). These assemblies were later on visualized on cryo-EM micrographs from where the structure for 12mers and 16-mers could be resolved (Supplementary Figure S4E, and Lizarrondo et al., 2021).

## Discussion

**FIGURE 9:**
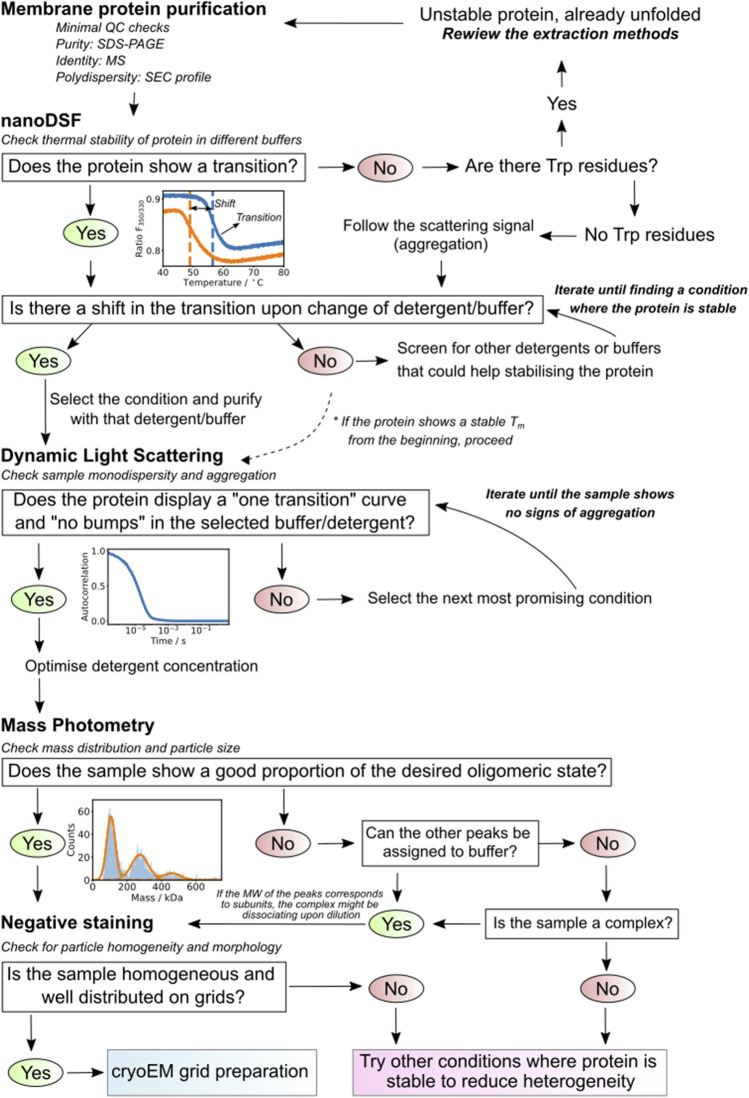
Summary diagram. The scheme presents a defined decision-making strategy based on the results of each of the used techniques. It guides the researcher on how to proceed depending on the results of the steps previously applied.

The presented biophysical pipeline allows us to efficiently optimize conditions for sample preparation for structural biology studies prior to electron microscopy, helping to reduce the costly and time-consuming cryo-EM screening of grids. DSF is used to optimize buffer conditions and to select the optimal detergent. Additionally, DLS gives information regarding the presence of large aggregates in the sample and can be used to identify the lowest possible detergent concentration preventing aggregation. It is crucial to monitor the onset of denaturation by DSF to minimize the presence of aggregates that can be detrimental for cryo-EM. Here, the limitation is that it does not account for those aggregates that arise upon vitrification, interaction with the grid, and at the air–water interface (Noble et al., 2018). Finally, mass photometry is used to check the size distribution, oligomeric states and integrity of complexes, providing information of molecular masses at the single particle level and their abundance in the sample (see Figure 9 for “decision making” after each step of the pipeline). Importantly, the eSPC platform offers tools to analyze and understand DSF and mass photometry experiments in a user-friendly webserver (spc.embl-hamburg.de). A detailed EM sample screening workflow is provided with instructions for performing negative stain and cryo-EM sample preparation for single particle analysis. Each biological system will require adjustments at different steps, and sample preparation still remains to be an iterative empirical procedure. The provided pipeline could be used as a guide and would help in the decision making when working with challenging samples. We are aware that most laboratories might not have all the equipment described in our pipeline. However, there are different opportunities offering trans-national access to cutting-edge biophysical infrastructures that only require a simple application and description of the project. Information regarding access to our facility and others can be provided upon request (contact us at spc@embl-hamburg.de).

## Data Availability

The original contributions presented in the study are included in the article/Supplementary Material; further inquiries can be directed to the corresponding author.
