# Supplementary material for: Biophysical Screening Pipeline for Cryo-EM Grid Preparation of Membrane Proteins
Source: Front Mol Biosci. 2022 Jun 23;9:882288. doi: 10.3389/fmolb.2022.882288 (PMC9259969; doi:10.3389/fmolb.2022.882288)
Supplement: Supplementary file 1 [file DataSheet1.pdf]

Supplementary information: Biophysical screening pipeline for cryo-EM grid preparation of membrane proteins

Table of contents:

1. IJ1 protein purification
2. TolC protein purification
3. hENTH protein purification
4. AENTH Supplementary Figure
5. PhotoMol User Documentation

1. IJ1 protein purification

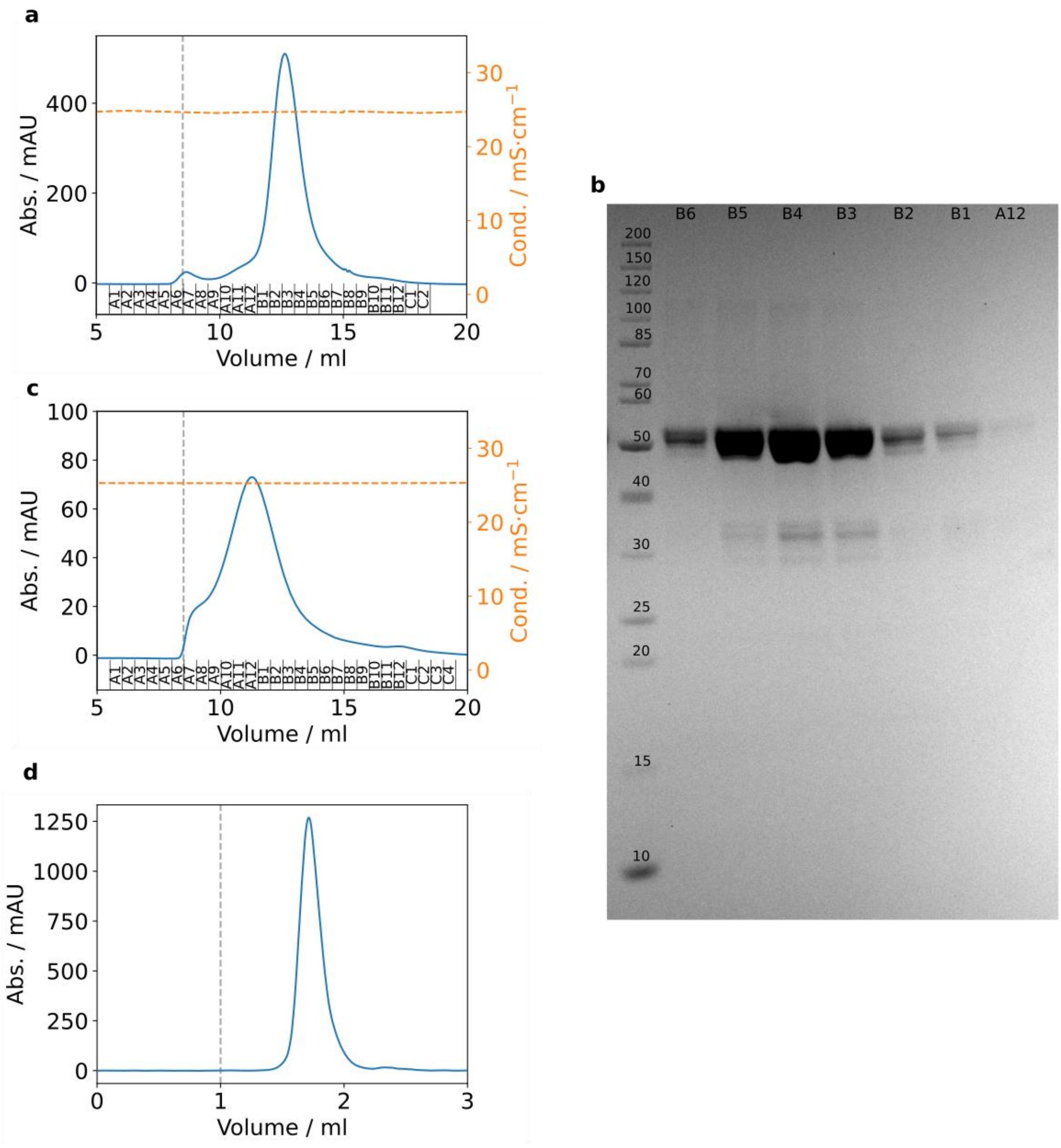

Fig. S1: Size exclusion chromatography of IJ1 solubilised with DDM (a, Superdex 200 10/300 column) and its respective SDS-PAGE gel (b). IJ1 sample solubilised in DDM was later subjected to size exclusion chromatography to perform a buffer exchange in LMNG (c, Superdex 200 10/300 column) and amphipol A8-35 (d, Superose 6 3.2/300 column). The elution volumes for the samples correlate with the size of the micelar-protein and amphipol-protein complexes. The size of an empty micelle in the current buffer is around 59.5 KDa for DDM and 93 KDa for LMNG. The void volumes V_0 of the columns are shown as grey dashed line in a, c, and d.

2. TolC protein purification

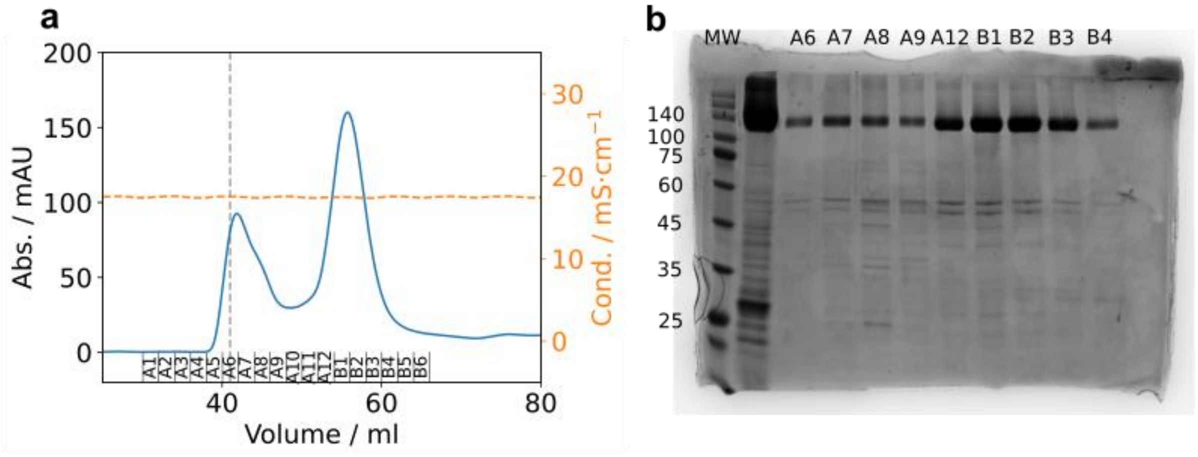

Fig. S2: Size exclusion chromatography of TolC (a) and SDS-PAGE gel of relevant fractions. The void volume V_0 of the column is shown as grey dashed line in (a).

3. hENTH protein purification

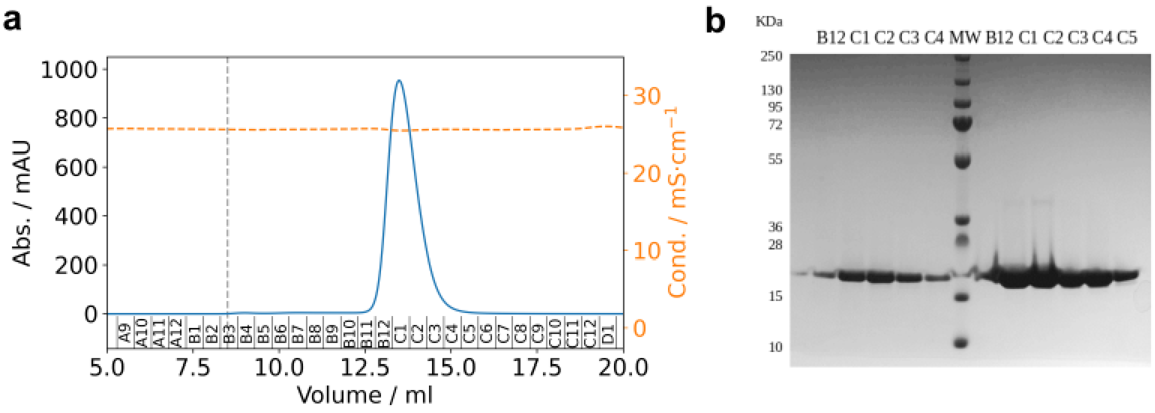

Fig. S3: Size exclusion chromatography of hENTH (a) and SDS-PAGE gel of relevant fractions. The void volume V_0 of the column is shown as grey dashed line in (a).

4. AENTH system: MP vs DLS and cryoEM

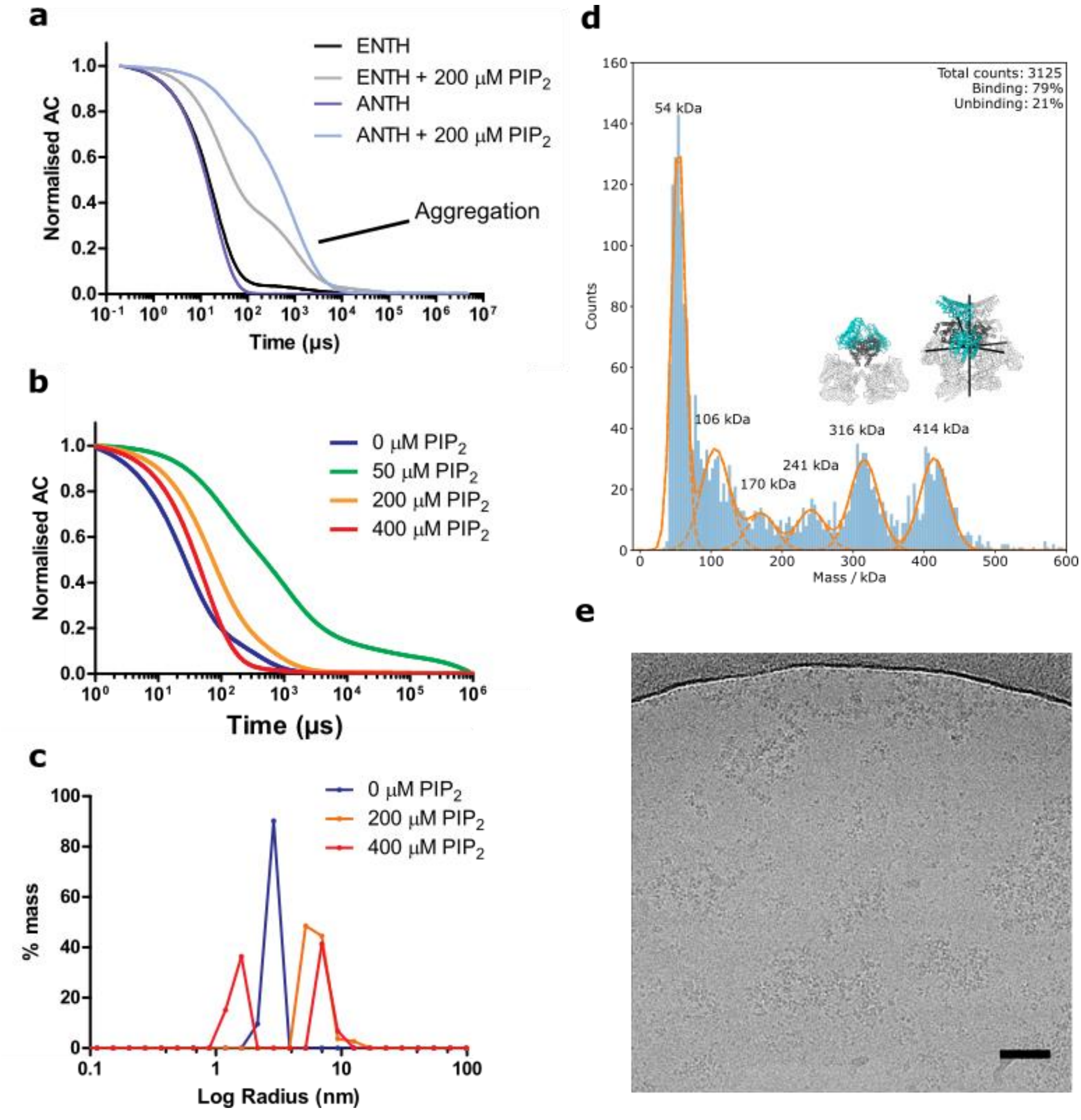

Fig. S4. Comparison of DLS vs MP. a) DLS autocorrelation curves for ANTH and ENTH domains from *S. cerevisiae* in presence and absence of 200 µM of PIP_2. The significant shift of the auto-correlation curve towards longer times and the presence of a bump are indicators of larger oligomers and/or aggregates present in the sample. b and c) Dynamic light scattering (DLS) of the ANTH and ENTH (*S. cerevisiae*) interaction in the absence of PIP_2 (blue); with 50 µM PIP_2 (green); 200 µM PIP_2 (orange) and 400 µM PIP_2 (red). b) Autocorrelation curves. The green curve is a typical example of a bad quality sample displaying macromolecular aggregation in this case due to the low concentration of phospholipids added to solubilize the forming complex. The red curve is a sample prepared above the critical micellar concentration (CMC) of PIP_2 which is around 200 µM. c) Intensity Mass distribution. A peak corresponding to PIP_2 micelles is observed below 2.5 nm (red line). d) Mass Photometry of a complex of

yeast AENTH complex in presence of 200 µM PIP_2. The peak distribution indicates the presence of different oligomeric states in the sample. The structure of the most prominent ones, a 12-mer and a 16-mer, is shown above the corresponding peaks. e) cryoEM electron micrograph of yeast AENTH complex in presence of 200 µM PIP_2. Several assemblies were detected on this sample whose structure is described in Lizarrondo et al., 2021. Scale bar is 50 nm.

4. PhotoMol User Documentation

April 2022

Table of Contents

1. Analysis

    1.1. Fitting model
    1.2. Input file
    1.3. Bin width
    1.4. Minimum observed mass
    1.5. Starting values
    1.6. Upper limit for the standard deviation
    1.7. Tolerance to the initial guesses
    1.8. Window range
    1.9. Baseline
    1.10. Curve fitting

2. Calibration

Overview

PhotoMol was developed to estimate the masses of different species in a sample after a Mass Photometry experiment. More details about this technology can be found at https://www.refeyn.com/.

1. Analysis

1.1. Fitting model

The function that we use to fit is a sum of truncated Gaussians

$$\hat{y} = b + \sum_{i=1}^{n} g_i(x) \qquad (1)$$

where *y* represents the histogram counts, *x* the masses, *n* is the number of truncated Gaussians *g(x)*, *b* is a user-defined baseline, and *g(x)* is defined as follows.

$$\boldsymbol{g(x) = amp * exp\left(-\frac{(x-center)^2}{2\sigma^2}\right)\ if\ x \geq x_{threshold}\ else\ 0} \qquad (2)$$

where $x_{threshold}$ is the minimum value of *x* (mass) that can be observed, *center* is the center of the gaussian, σ is the standard deviation, and *amp* is the amplitude.

1.2. Input file

PhotoMol accepts as input a '.h5' (Hierarchical Data Format) file. This file should have one 1D dataset called 'masses_kDa' and can be exported using the software Refeyn DiscoverMP. In the DiscoverMP version < 2.5, the file eventsFitted.h5 is saved in the folder when saving the results. In version 2.5 the events can be exported individually selecting a custom file name.

Additionally, a csv (comma-separated values) file with headers can be loaded. The column 'masses_kDa' and 'contrasts' are respectively required for the mass distribution data analysis and calibration.

1.3. Bin width

Integer value (kDa) used to group data and build the histogram.

1.4. Minimum observed mass

Integer value (kDa) that defines the left limit for the truncated multi gaussian.

1.5. Starting values

List of numbers separated by spaces. Each value is used to define the initial guess of the mean of a (truncated) Gaussian.

1.6. Upper limit for the standard deviation

Integer value (kDa) used to calculate fitting boundaries for the gaussian deviations.

1.7. Tolerance to the initial guesses

Integer value (kDa) used to calculate fitting boundaries for the gaussian means.

1.8. Window range

Set the limits (kDa) for constructing the histogram.

1.9. Baseline

Integer value used in Equation 1 (parameter *b*). Useful when there is constant noise.

1.10 Curve fitting

The histogram defined by the bin width and window region is fitted using the Levenberg Marquardt (damped least-squares) algorithm.

2. Calibration

Ratiometric contrasts can be converted to masses by loading a '.h5' file with known masses (3 different species at least), or using parameters from a previous calibration. In both cases, the calibration experiment should have been done with the same buffer, at the same temperature, and using the same instrument parameters (i.e., the field of view).

The fitting function and parameters are the same as previously described for analyzing the histogram of the observed masses, with the exception that the units are now ‘Ratiometric contrasts’ (instead of kDa).

Packages

PhotoMol is possible thanks to:

R language: R Core Team (2020). R: A language and environment for statistical computing. R Foundation for Statistical Computing, Vienna, Austria. URL https://www.R-project.org/.

R package shiny: Winston Chang, Joe Cheng, JJ Allaire, Yihui Xie and Jonathan McPherson (2020). shiny: Web Application Framework for R. R package version 1.4.0.2. https://CRAN.R-project.org/package=shiny

R package shinydashboard: Winston Chang and Barbara Borges Ribeiro (2018). shinydashboard: Create Dashboards with 'Shiny'. R package version 0.7.1. https://CRAN.R-project.org/package=shinydashboard

R package ggplot2: H. Wickham. ggplot2: Elegant Graphics for Data Analysis. Springer-Verlag New York, 2016.

R package reshape2: Hadley Wickham (2007). Reshaping Data with the reshape Package. Journal of Statistical Software, 21(12), 1-20. URL http://www.jstatsoft.org/v21/i12/.

R package tippy: John Coene (2018). tippy: Add Tooltips to 'R markdown' Documents or 'Shiny' Apps. R package version 0.0.1. https://CRAN.R-project.org/package=tippy

R package shinyalert: Pretty Popup Messages (Modals) in 'Shiny'. R package version 1.1. https://CRAN.R-project.org/package=shinyalert

R package plotly: C. Sievert. Interactive Web-Based Data Visualization with R, plotly, and shiny. Chapman and Hall/CRC Florida, 2020.

R package shinyjs: Dean Attali (2020). shinyjs: Easily Improve the User Experience of Your Shiny Apps in Seconds. R package version 1.1. https://CRAN.R-project.org/package=shinyjs

R package reticulate: Kevin Ushey, JJ Allaire and Yuan Tang (2020). reticulate: Interface to 'Python'. R package version 1.16. https://CRAN.R-project.org/package=reticulate

R package shinycssloaders: Andras Sali and Dean Attali (2020). shinycssloaders: Add CSS Loading Animations to 'shiny' Outputs. R package version 0.3. https://CRAN.R-project.org/package=shinycssloaders

Python3.7 language: Van Rossum, G., & Drake, F. L. (2009). Python 3 Reference Manual. Scotts Valley, CA: CreateSpace.

Python package numpy: Travis E, Oliphant. A guide to NumPy, USA: Trelgol Publishing, (2006). Stéfan van der Walt, S. Chris Colbert, and Gaël Varoquaux. The NumPy Array: A Structure for Efficient Numerical Computation, Computing in Science & Engineering, 13, 22-30 (2011), DOI:10.1109/MCSE.2011.37

Python package pandas: Wes McKinney. Data Structures for Statistical Computing in Python, Proceedings of the 9th Python in Science Conference, 51-56 (2010)

Python package scipy: Pauli Virtanen, Ralf Gommers, Travis E. Oliphant, Matt Haberland, Tyler Reddy, David Cournapeau, Evgeni Burovski, Pearu Peterson, Warren Weckesser, Jonathan Bright, Stéfan J. van der Walt, Matthew Brett, Joshua Wilson, K. Jarrod Millman, Nikolay Mayorov, Andrew R. J. Nelson, Eric Jones, Robert Kern, Eric Larson, CJ Carey, İlhan Polat, Yu Feng, Eric W. Moore, Jake VanderPlas, Denis Laxalde, Josef Perktold, Robert Cimrman, Ian Henriksen, E.A. Quintero, Charles R Harris, Anne M. Archibald, Antônio H. Ribeiro, Fabian Pedregosa, Paul van Mulbregt, and SciPy 1.0 Contributors. (2020) SciPy 1.0: Fundamental Algorithms for Scientific Computing in Python. Nature Methods, 17(3), 261-272.
